# Mesenchymal Stromal Cell-Derived Extracellular Vesicles in Lung Diseases: Current Status and Perspectives

**DOI:** 10.3389/fcell.2021.600711

**Published:** 2021-02-15

**Authors:** Soraia C. Abreu, Miquéias Lopes-Pacheco, Daniel J. Weiss, Patricia R. M. Rocco

**Affiliations:** ^1^Laboratory of Pulmonary Investigation, Carlos Chagas Filho Biophysics Institute, Federal University of Rio de Janeiro, Rio de Janeiro, Brazil; ^2^National Institute of Science and Technology for Regenerative Medicine, Rio de Janeiro, Brazil; ^3^Biosystems & Integrative Sciences Institute, Faculty of Sciences, University of Lisbon, Lisbon, Portugal; ^4^Department of Medicine, College of Medicine, University of Vermont Larner, Burlington, VT, United States

**Keywords:** biomarkers, cell therapy, extracellular vesicles, inflammation, remodeling, respiratory disease, animal models

## Abstract

Extracellular vesicles (EVs) have emerged as a potential therapy for several diseases. These plasma membrane-derived fragments are released constitutively by virtually all cell types—including mesenchymal stromal cells (MSCs)—under stimulation or following cell-to-cell interaction, which leads to activation or inhibition of distinct signaling pathways. Based on their size, intracellular origin, and secretion pathway, EVs have been grouped into three main populations: exosomes, microvesicles (or microparticles), and apoptotic bodies. Several molecules can be found inside MSC-derived EVs, including proteins, lipids, mRNA, microRNAs, DNAs, as well as organelles that can be transferred to damaged recipient cells, thus contributing to the reparative process and promoting relevant anti-inflammatory/resolutive actions. Indeed, the paracrine/endocrine actions induced by MSC-derived EVs have demonstrated therapeutic potential to mitigate or even reverse tissue damage, thus raising interest in the regenerative medicine field, particularly for lung diseases. In this review, we summarize the main features of EVs and the current understanding of the mechanisms of action of MSC-derived EVs in several lung diseases, such as chronic obstructive pulmonary disease (COPD), pulmonary infections [including coronavirus disease 2019 (COVID-19)], asthma, acute respiratory distress syndrome (ARDS), idiopathic pulmonary fibrosis (IPF), and cystic fibrosis (CF), among others. Finally, we list a number of limitations associated with this therapeutic strategy that must be overcome in order to translate effective EV-based therapies into clinical practice.

## Introduction

Mesenchymal stromal cells (MSCs) have been widely studied for their potential regenerative and immunomodulatory actions in several preclinical experimental models and in early stage clinical trials of lung diseases (Lalu et al., 2012; Cruz and Rocco, 2020; Lopes-Pacheco et al., 2020). Although their mechanisms of action have yet to be fully elucidated, MSCs administered either locally or systemically have the ability to release a mix of bioactive molecules (Phinney et al., 2015; Cruz and Rocco, 2020; Lopes-Pacheco et al., 2020), which promote activation of endogenous repair pathways (Lai et al., 2015) and reprogramming of immune cells, leading to modulation of both inflammatory and remodeling processes (Bell et al., 2012; Cruz and Rocco, 2020; Lopes-Pacheco et al., 2020). The MSC secretome consists of an extensive mix of bioactive molecules that coordinate processes within the inflammatory microenvironment, influencing tissue regeneration (Zhao et al., 2010). These molecules include growth factors, cytokines/chemokines, and extracellular vesicles (EVs) that promote inhibition of cellular apoptosis and disorganized deposition of extracellular matrix and/or modulation of immune responses (Waszak et al., 2012; Phinney et al., 2015; Mohammadipoor et al., 2018; de Castro et al., 2019; Popowski et al., 2020).

The conditioned medium (CM) of MSCs yields bioactive products from which EVs can be isolated. Notably, administration of MSC-derived CM can promote protective actions similar to those of the parent cells, which suggests that secretion of trophic factors might be the key mechanism underlying the therapeutic effects of MSCs (Lai et al., 2015; Mohammadipoor et al., 2018). Furthermore, administration of MSC secretome-derived bioactive products, including EVs, is associated with additional safety advantages compared to living cells, since EVs do not carry the potential risks of whole-cell transplants. MSC secretome products are non-oncogenic and less immunogenic than living cells, can be administered intravascularly without the risk of causing clots, and can be sterilized, handled, and stored more easily, with no need for cryoprotectants (e.g., dimethyl sulfoxide) (Goolaerts et al., 2014; Giebel et al., 2017; Gimona et al., 2017). Therefore, MSC secretome-derived bioactive products have become an emerging therapeutic option to replace the administration of MSCs as potent therapies for different lung disorders.

Several studies have demonstrated that EVs act on cell-to-cell communication and, consequently, exert a relevant role in the paracrine/endocrine actions induced by MSC-based therapy. According to the minimal criteria established by the International Society for Extracellular Vesicles (ISEV), EVs are small spherical membrane fragments that can be classified into three different types: exosomes, microvesicles (MVs) or microparticles, and apoptotic bodies (Theìry et al., 2018). Essentially every cell type can secrete EVs, although their cargo differs significantly. In fact, EVs can modify the fate and phenotype of recipient cells (Camussi et al., 2010). Additionally, EVs can also be harvested *in vivo* from several body fluids such as blood, breast milk, bronchoalveolar lavage fluid (BALF), serum, and even urine (Almqvist et al., 2008; Baglio et al., 2012). Therefore, persistent circulating EVs in biological fluids can serve as an indicator for the diagnosis of several diseases, providing significant information regarding individual pathological and physiological status (Eissa, 2013; Asef et al., 2018).

Many parameters have been used for EV characterization. These include biogenesis pathway, flotation density on a sucrose gradient, ionic composition, lipid composition, protein cargo, and sedimentation rate and size, among others. It is important to note that none of these parameters is definitive or exclusive to any specific type of EV (Theìry et al., 2018); therefore, they should be well-documented to ensure reproducibility. Ideally, EVs should be characterized by quantifiable physical characteristics, biochemical composition, and functional assays (Witwer et al., 2019). Our knowledge regarding EVs is continuously expanding, but many questions remain unanswered. In this review, we describe the main characteristics of different EVs with a particular focus on MSC-derived EVs. We also outline the state of the science regarding the potential of these bioactive products as therapy for various lung diseases. Finally, we summarize the key advantages and limitations that should be considered in order to translate effective EV-based therapies into the clinical scenario.

## Extracellular Vesicles

### Biogenesis and Classification of Extracellular Vesicles

**FIGURE 1:**
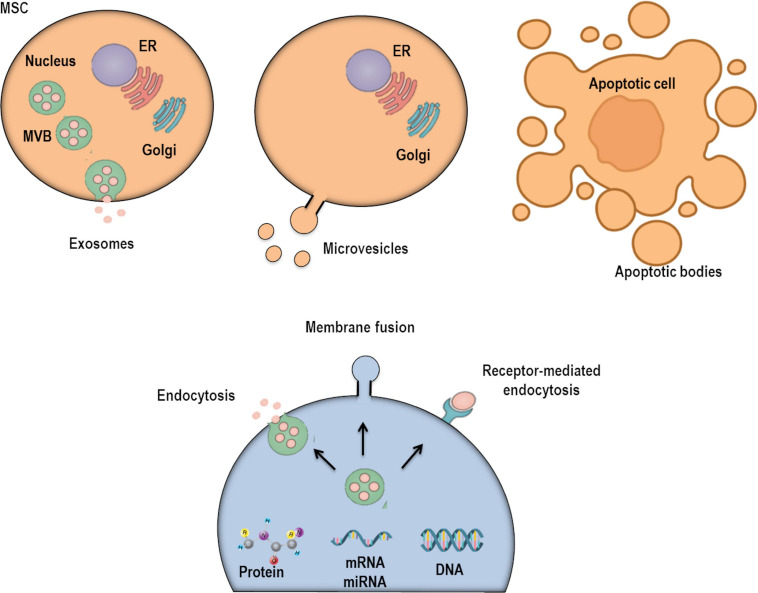
Extracellular vesicles (EVs) are currently classified into three subpopulations depending on their subcellular origin, secretion mechanism, and size: exosomes, microvesicles, and apoptotic bodies. There are several mechanisms through which EVs may interact with recipient/target cells: interactions with plasma membrane (PM) receptors, internalization into endocytic compartments, and fusion with PM.

Extracellular vesicles are plasma membrane-derived structures, ranging from 30 nm to 5 μm in diameter, that are limited by a phospholipid bilayer. These structures comprise a heterogeneous population of vesicles that contain various bioactive molecules (proteins, lipids, DNA, mRNA, and microRNAs), which confer differences in their biological activities (Theìry et al., 2018; Witwer et al., 2019) (Figure 1). Eirin et al. (2014) found almost 400 miRNAs analyzed in MSC-derived EVs, and the levels of four were significantly higher in EVs compared to parent MSCs: miR-148a, miR-378, miR-532-5p, and let-7f. Additionally, a significant difference was found between the protein levels expressed in MSCs and those measured in their EVs. These include proteins associated with angiogenesis, apoptosis, blood coagulation, extracellular matrix remodeling, and inflammation, which demonstrated a higher expression in EVs compared to their parent MSCs (Eirin et al., 2014). Nevertheless, further studies are necessary to determine whether these differences between the biological activity and content of EVs and their parent MSCs are indeed responsible for the distinct therapeutic action of EVs.

Based on the minimal criteria for definition, characterization, and studies of EVs (Theìry et al., 2018; Witwer et al., 2019), cells are able to produce and secrete three main types of EVs: exosomes, MVs, and apoptotic bodies. These are grouped based on their cellular origin, secretion pathway, and size (Figure 1). Exosomes were observed for the first time in reticulocytes of rodents (Harding et al., 1983) and sheep (Pan et al., 1985) in the 1980s. These structures are nano-sized vesicles (30–150 nm) with a homogeneous shape (Cocucci et al., 2009), derived from specialized compartments—the late endosomes or multivesicular bodies (MVBs)—and released into the extracellular compartment by exocytosis (Trajkovic et al., 2008; Henne et al., 2013; Kowal et al., 2014; Guiot et al., 2019). They are released constitutively from cells by chemical and physical stimuli: soluble factors and shear stress, respectively (Kinoshita et al., 2017). During exosome biogenesis, the limiting membrane of the MVB buds inward, forming intraluminal vesicles (ILVs). These structures are then released into the extracellular environment as exosomes after fusion with the plasma membrane. This process is intermediated by p53-regulated exocytosis, which is dependent on cytoskeletal activation, although independent of calcium influx (Hessvik and Llorente, 2018). The endosomal sorting complex required for transport (ESCRT) is also necessary for ILV delivery into MVBs (Hessvik and Llorente, 2018). MVBs can then take two pathways: (1) fuse with the plasma membrane by using RAB GTPases in an ESCRT-independent mechanism or (2) fuse with lysosomes for cargo degradation (Frydrychowicz et al., 2015; Fujita et al., 2015). Importantly, during the exocytosis of this vesicle, it can integrate multiple proteins from the plasma membrane: tetraspanins, membrane transporters, ion channels, fusion proteins (CD9, CD63, CD81, and CD82), Tsg10, raft proteins, major histocompatibility complex (MHC) proteins, and targeting/adhesion molecules (Gon et al., 2020). This suggests that the composition of the exosomal limiting membrane can vary depending on cellular origin or activation of intracellular signaling pathways. MVs are structures ranging from 0.1 to 1 μm in diameter, which originate from outward budding and scission of the plasma membrane (Simoncini et al., 2009). Their biogenesis begins with translocation of phosphatidylserine into the outer membrane leaflet (Cocucci et al., 2009). Thereafter, a contraction of cytoskeletal structures occurs, releasing the nascent MVs into the extracellular compartment (Cocucci et al., 2009). Notably, a wide range of plasma membrane proteins has been associated with the process of MV scission, including members of the ADP-ribosylation factor small G-protein subfamily, ARF1 and ARF6, and TSG101 (a component of the ESCRT) (Kinoshita et al., 2017). MVs are also enriched in ceramide and sphingomyelin and have high levels of proteins associated with lipid rafts, cholesterol, and phosphatidylserine. Furthermore, the biological activities of MVs are largely variable, as their bioactive cargo is dependent on cellular origin and stimuli that can alter intracellular signaling (Kinoshita et al., 2017). Once these particles are released in the extracellular compartment, they can affect the microenvironment by altering the phenotype of recipient cells (Cocucci et al., 2009). Finally, apoptotic bodies are larger EVs (1–5 μm in size) derived from dying cells destined to be cleared by phagocytic cells (Kerr et al., 1972; Atkin-Smith et al., 2015; Theìry et al., 2018). These vesicles are characterized by exposing phosphatidylserine to the extracellular compartment and containing histones and fragmented DNA (Kerr et al., 1972; Atkin-Smith et al., 2015; O’Farrell and Yang, 2019). They can also contain cell organelles and non-coding RNAs. Indeed, recent studies have suggested that recipient cells might obtain certain genetic information by engulfing apoptotic bodies (Galleu et al., 2017; Abreu et al., 2019; O’Farrell and Yang, 2019).

Isolating pure EV populations is difficult because different EV types can overlap in size and density (Theìry et al., 2018; Witwer et al., 2019). In fact, a preparation containing EVs < 200 nm can be called as “small EVs,” whereas EVs > 200 nm are called “medium or large EVs” (Theìry et al., 2018). However, this definition is based on EV size; exosomes, MVs, and apoptotic bodies differ according to their biogenesis pathway (Witwer et al., 2019). Therefore, establishment of standard methods that can efficiently enable the separation of a subpopulation of EVs remains a major challenge.

As mentioned above, EV contents are composed of different types of nucleic acids, proteins, and lipids; however, certain organellar contents (e.g., mitochondria) can also be transported within MVs and apoptotic bodies. Notably, EV cargo and certain biological activities may reflect, at least partially, features of their cellular origin (Chen J. et al., 2014), as well as their mode of biogenesis and exogenous stimuli. Two major databases store information regarding EVs: (1) the VESICLEPEDIA^1^ collects information on different EV contents, while (2) ExoCarta^2^ collects information on the exosomes derived from different cell types and organisms.

Extracellular vesicles exert a critical role in the intercellular compartment through distinct mechanisms. These include direct binding to surface receptors, inhibition/activation of intracellular pathways, horizontal transfer of genetic information, and protein and lipid delivery (Tetta et al., 2011). Due to the remarkable ability of EVs to modify the fate and phenotype of recipient cells (Akyurekli et al., 2015), they have become a promising therapeutic option. In particular, MSC-derived EVs have demonstrated, in multiple *in vitro* and *in vivo* models, the potential to circumvent some of the safety risks related to whole-cell transplantation. Nevertheless, further insights on the cargo of EVs from different origins and how these can be modulated to enhance tissue regeneration are still required (Baglio et al., 2012; Akyurekli et al., 2015).

### Methods for Extracellular Vesicle Isolation

Several methods have been employed to isolate EVs, each of which has its own benefits and disadvantages. Conventional methods include the measurement of EV size and flotation density through gel filtration, microfiltration, and ultracentrifugation (the most commonly used technique) (Thery et al., 2006; Tauro et al., 2012; Taylor and Shah, 2015; Theìry et al., 2018; Witwer et al., 2019). Sequential centrifugation steps (Mendt et al., 2018) and ultrafiltration are methods for large-scale EV production (Bari et al., 2019). Additional methods based on EV solubility have emerged, including precipitation by using polyethylene glycol, protamine, or sodium acetate (Deregibus et al., 2016; Gámez-Valero et al., 2016). Some methods based on interactions with molecules on the EV surface have also been investigated (Taylor and Shah, 2015; Konoshenko et al., 2018). In short, the best EV isolation technique depends on time and cost, and different techniques may result in different products with different therapeutic effects.

### Characterization of Extracellular Vesicles

The ISEV recommends that EV characterization should be carried out using biomarkers from different categories, which include cytosolic and transmembrane proteins (Camussi et al., 2010; Lötvall et al., 2014). To further investigate population heterogeneity, the ISEV recommends the use of two additional methods, such as single-particle tracking methods and electron microscopy (Camussi et al., 2010; Lötvall et al., 2014; Witwer et al., 2017; Theìry et al., 2018; Witwer et al., 2019).

### Measurement of Extracellular Vesicle Size

It is important to note that samples will always be composed of different types of EVs, regardless of which method is employed to isolate and separate them. Therefore, measurement of EV count and size is useful to ensure similarity across studies. Some commercially available particle analyzers have been frequently used to carry out these measurements. NanoSight (NanoSight, Amesbury, United Kingdom) can visualize and measure particle size, number, and concentration in real time. Zetaview (Particle Metrix, Germany) is an alternative that can measure hydrodynamic particle size, zeta potential, and concentration. The qNano system (Izon Sciences Ltd., Palmerston North, New Zealand) can measure EV amount and size by using a tunable resistive pulse-sensing technology.

### Biological Activities and Potential Mechanisms of Action of Extracellular Vesicles

There are several mechanisms by which EVs may interact with recipient cells. These include plasma membrane receptors, fusion of EVs with the plasma membrane itself, and internalization into endocytic compartments (Phinney et al., 2015). After interaction with recipient cells, EVs may release their cargo in the cytosol, leading to inhibition or activation of certain signaling pathways. A subset of mRNAs and miRNAs present in EV cargo may also alter gene expression of recipient cells, leading to modulation of biological activities (Table 1). Furthermore, EVs may induce reprogramming and alter the phenotype and/or behavior of recipient cells (Camussi et al., 2013). Nevertheless, the exchange of vesicle cargo can occur in a two-way fashion, i.e., from MSCs to damaged cells or from a harmful microenvironment to MSCs. In this context, Dooner et al. (2008) reported that bone marrow-derived MSCs started to express genes for lung-specific proteins when cocultured with injured lung cells; these proteins include club cell-specific components as well as surfactant proteins. This phenomenon may be attributed to the EV-mediated transfer of lung-specific mRNAs from the damaged lung cells to bone marrow cells (Dooner et al., 2008).

**TABLE 1:** Biological activity of microRNAs derived from MSC-EVs in respiratory diseases.

**References**	**MicroRNA**	**Function**
Song et al., 2017	miR-146a	Polarization of macrophages to M2 anti-inflammatory phenotype
Wu et al., 2018	miR-126	↓ Sprouty-related, EVH1 domain-containing protein 1 (Spread-1), ↑ Endothelial cell function
Hao et al., 2019	miR-145	Antimicrobial activity *in vivo*, ↓ Multidrug resistance-associated protein 1 activity
Li et al., 2019	miR-21-5p	↓ lung cell apoptosis
Yi et al., 2019	miR-30b-3p	↑ Proliferation and ↓ apoptosis of alveolar epithelial cells
Zhou et al., 2019	miR-126	↓ VEGFα and HMGB1 levels, ↑ tight junction protein expression
Chen et al., 2020	miR-100	↓ Inflammation and apoptosis Downregulation of mTOR signaling
Fang et al., 2020	miR-146a-5p	Inhibition of group 2 innate lymphoid cells (ILC2s)
Wang et al., 2020	miR-27a-3p	↓ TNF-α levels, Polarization of macrophages to M2 anti-inflammatory phenotype
Zhang C. et al., 2020	miR-191	Inhibition of bone marrow morphogenetic protein receptor 2

*HMGB, high mobility group box; mTOR, mammalian target of rapamycin; VEGF, vascular endothelial growth factor.*

**FIGURE 2:**
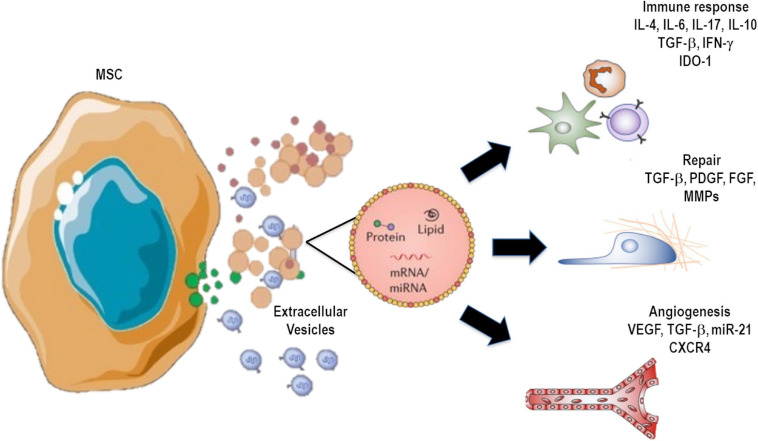
Extracellular vesicle (EV) cargo can contain several molecules, including proteins, mRNA, microRNAs, DNAs, and lipids, as well as organelles that can be transferred to recipient cells, inducing modulation of immune responses and remodeling processes.

Human MSC-derived EVs have been more widely studied in the last few years (Gomzikova et al., 2019; Gowen et al., 2020; Zhang B. et al., 2020). These vesicles have a cargo composed of ribonucleoproteins that are involved in the synthesis and intracellular trafficking of RNAs, suggesting a distribution and dynamic rearrangement of RNA related to cell differentiation, development, and repair—all of which can contribute to a more efficient recovery process after damage (Figure 2) (Collino et al., 2010). On the other hand, mounting evidence indicates that MSC-derived EVs are able to promote immunomodulatory actions. These include inhibition of immune cell differentiation, polarization of macrophages, expansion of regulatory T cells, as well as production and secretion of anti-inflammatory and resolutive mediators (Mokarizadeh et al., 2012; de Castro et al., 2019) (Figure 2). In this context, MSCs or their EVs have been shown to promote macrophage polarization toward an M2 anti-inflammatory profile rather than the conventional M1 pro-inflammatory phenotype, which can also contribute to inflammation resolution and tissue repair (Zhang et al., 2014; Abreu et al., 2018; Mansouri et al., 2019). A specific inflammatory environment can stimulate MSCs to produce and secrete subsets of EVs that are able to act on endogenous repair pathways and modulate immune responses (Hofmann et al., 2012; Tomasoni et al., 2013). Eirin et al. (2014) found that MSC-derived EVs overexpress certain transcription factor mRNAs, such as *MDFIC*, *NRIP1*, and *POU3F1.* Genes associated with adipogenesis (*CEBPA* and *KLF7*) and angiogenesis (*HES1*, *HGF*, and *TCF4*) were also found to be upregulated (Eirin et al., 2014).

Extracellular vesicles derived from immune or damaged cells may stimulate differentiation of resident stem cells or recruit stem-like cells present in other organs in order to promote a more efficient tissue repair (Bruno and Camussi, 2013). EVs can also exert immunomodulatory actions depending on their cellular origin or external stimulation (Bourdonnay et al., 2015; de Castro et al., 2019). For example, mycobacteria-infected alveolar macrophages are known to release vesicles containing pathogen-derived pro-inflammatory molecules that upregulate heat-shock protein 70, which then stimulates the nuclear factor kappa B (NF-κB) pathway by promoting activation of toll-like receptors (TLRs) and downstream signaling (Katsuda et al., 2013; Bruno et al., 2015; Fujita et al., 2015). Furthermore, EVs derived from lipopolysaccharide (LPS)-activated monocytes have been shown to induce apoptosis of recipient cells by transfer and activation of the caspase-1 cascade (Sarkar et al., 2009). On the other hand, EVs secreted by dendritic cells are able to promote humoral responses against antigens processed by these cells before EV purification, which yields potent protection against infections (Qazi et al., 2010; Bruno et al., 2015).

### Extracellular Vesicle-Secreting Cells in the Respiratory Tract

Extracellular vesicles can be produced and secreted by virtually all cell types in the respiratory system. An increasing number of studies have characterized EVs from alveolar macrophages, bronchial epithelial cells (BECs), fibroblasts, type II pneumocytes, and vascular endothelial cells under normal physiological or pathological conditions (Kulshreshtha et al., 2013). In this context, BECs have been considered a main source of pulmonary EVs (Kulshreshtha et al., 2013); release can be stimulated by environmental components, such as cigarette smoke, which induce injury of these cells and promote their release of pro-inflammatory cytokines and chemokines (Kadota et al., 2016). BECs are able to secrete EVs of various sizes, which can contain a wide range of cargoes such as membrane-tethered mucins (MUC)-1, -4, and -6 (Kesimer et al., 2009; Kesimer and Gupta, 2015). These components are present on the surface of BEC-derived EVs and can neutralize human influenza A viruses (Kesimer et al., 2009). One study in a mouse model of ovalbumin-induced allergic asthma found a twofold increase in counts of immune cell-derived EVs and increased expression of immune cell-derived miRNAs (including miR-142a and miR-223) after allergy induction (Pua et al., 2019). By using a cell type-specific membrane marker, the authors found that the majority of EVs released into BALF were derived from airway-lining epithelium (Pua et al., 2019).

Alveolar macrophages are also major sources of EVs (Kulshreshtha et al., 2013). Macrophage derived-EVs can induce differentiation of other macrophages through miRNA-223 transfer (Ismail et al., 2013). Procoagulant EVs can also be released by macrophages in response to cigarette smoke (Li et al., 2010), upregulating the production of pro-inflammatory mediators by lung epithelial cells. Intercellular adhesion molecule-1, interleukin-8, and monocyte chemotactic protein-1 are some important mediators that were observed to be upregulated by cigarette smoke exposure (Cordazzo et al., 2014).

Vascular endothelial cells can produce and secrete EVs that participate in several biological processes, depending on their cargo. These processes include angiogenesis, coagulation, endothelial function, and inflammation (Kadota et al., 2016). EVs may activate fibroblasts and stimulate their differentiation into myofibroblasts, contributing to excessive deposition of extracellular matrix components, or prevent the extensive activation of these cells and thus maintain homeostasis (Lacy et al., 2019). B cells are able to secrete EVs that activate or inhibit several immune cell populations (Raposo et al., 1996). Furthermore, EVs secreted by dendritic cells appear to modulate immune reactions by activating T and B cells, suggesting intercellular communications to modulate airway inflammation and allergic reactions.

## Extracellular Vesicles in Lung Disease Pathogenesis and Therapy

Although the effects of MSC therapy in experimental models of lung diseases have been extensively investigated (Lopes-Pacheco et al., 2016, 2020; Cruz and Rocco, 2020), the study of EVs as a possible alternative strategy to MSC administration is less well explored, and many questions still need to be further elucidated (Table 2). Nevertheless, a growing number of experimental studies have investigated the potential of MSC-derived EVs as a therapy for lung diseases. In this section, we summarize some important findings from experimental research of EV-based therapies for chronic obstructive pulmonary disease (COPD), pulmonary infections [including coronavirus disease 2019 (COVID-19)], asthma, acute respiratory distress syndrome (ARDS), idiopathic pulmonary fibrosis (IPF), and cystic fibrosis (CF), among others.

**TABLE 2:** Studies evaluating effects of administration of mesenchymal stromal cell-derived extracellular vesicles in models of lung diseases.

**References**	**Injury model**	**Therapy**	**Route**	**Regimen**	**Main outcomes**
Zhu et al., 2014	*E. coli*-induced ARDS	Double amount of MVs from 1.5 × 10^6^ BM-MSCs	i.t.	12 h after injury	↓ Extravascular lung water, protein permeability, lung inflammation, pro-inflammatory mediators (MIP-2, TNF-α) Therapeutic effects were partially exerted by KGF transfer
Cruz et al., 2015	*Aspergillus fumigatus* hyphal extract-induced allergic asthma	EVs from 1 × 10^6^ BM-MSCs	i.v.	Immediately after final challenge with *Aspergillus fumigatus* hyphal extract	↓ Airway hyperreactivity, lung inflammation, level of pro-inflammatory mediators (IL-17, IL-4, IL-5, IL-12-p40, KC, RANTES)
Hayes et al., 2015	VILI	CM from 4 × 10^6^ BM-MSCs	i.v.	∼3 h after initiation of VILI	MSCs were more effective than CM in improving lung function and inflammation
Monsel et al., 2015	*E. coli*-induced ARDS	MVs from 1 × 10^6^ BM-MSCs	i.t.	4 h after injury	↑ Survival, phagocytosis activity, ↓ Lung inflammation, interstitial congestion, protein permeability, bacterial loading
Phinney et al., 2015	Silicosis	∼3 × 10^11^ exosomes from BM-MSCs	i.v.	3 days after injury	MSC transfer mitochondria to macrophages and lung tissue, ↑ Macrophage bioenergetics Inhibition of TLR signaling in macrophages
de Castro et al., 2017	Ovalbumin-induced allergic asthma	EVs from 1 × 10^6^ AD-MSCs	i.v.	1 day after final challenge with ovalbumin	↑ Lung function, ↓ Lung inflammation and fibrosis level of pro-inflammatory mediators (IL-4, IL-5, eotaxin)
Kim H. J. et al., 2017	Elastase-induced emphysema	Artificial nanovesicles from AD-MSCs	i.t.	7 days after injury	↑ Proliferative capacity of an alveolar epithelial cell line, FGF2 signaling, ↓ Mean linear intercept
Morrison et al., 2017	LPS-induced ARDS	EVs from 7.5 × 10^6^ BM-MSCs	i.n.	4 h after injury	Macrophage polarization to M2-like phenotype ↑ Phagocytosis activity, ↓ Lung inflammation, level of pro-inflammatory mediators (IL-8, TNF-α)
Song et al., 2017	CLP-induced polymicrobial sepsis	Exosomes from 1 × 10^6^ UC-MSCs (primed or not with IL-1β)	i.v.	4 h after injury	↑ Survival, macrophage polarization to M2-like phenotype, exosomal miR-146a, ↓ Lung inflammation
Tang et al., 2017	LPS-induced ARDS	MVs from 1 × 10^6^ BM-MSCs	i.t.	Simultaneously to injury induction	↓ Lung inflammation and injury, protein permeability, MIP-2 level, Therapeutic effects were partially exerted by Ang-1 expression
Braun et al., 2018	Hyperoxia-induced bronchopulmonary dysplasia	3.4 × 10^9^ exosomes from BM-MSCs	i.p.	Simultaneously to injury induction	↓ Lung damage, ↑ VEGF levels Improve pulmonary vasculature
Chaubey et al., 2018	Hyperoxia-induced bronchopulmonary dysplasia	Exosomes from UC-MSCs	i.p.	48 h after injury	↓ Lung inflammation, pulmonary arterial hypertension Therapeutic actions were partially modulated by TSG-6
Khatri et al., 2018	Influenza virus-induced ARDS	EVs from BM-MSCs	i.n.	12 h after injury	↓ Lung inflammation, virus replication, levels of pro-inflammatory mediators (TNF-α, CXCL10)
Willis et al., 2018	Hyperoxia-induced bronchopulmonary dysplasia	Exosomes from WJ-MSCs or BM-MSCs	i.v.	Day 7 of hyperoxia protocol	↑ Lung function, ↓ Lung architecture distortion, fibrosis, pulmonary hypertension and vascular remodeling
Hao et al., 2019	*E. coli*-induced ARDS	1 × 10^10^ exosomes from BM-MSCs	i.t.	4 h after injury	miR-145 transfer, ↓ MRP1 expression, ↑ LTB_4_ production, bacterial clearance
Li et al., 2019	Lung ischemia/reperfusion	Exosomes from BM-MSCs	i.t.	16 h before ischemia protocol	Exosome transport of anti-apoptotic miR-21-5p, ↓ Lung edema, M1 polarization, level of pro-inflammatory mediators (HMGB1, IL-17, IL-1β, IL-6, IL-8, and TNF-α)
Mansouri et al., 2019	Bleomycin-induced lung fibrosis	Exosomes from BM-MSCs	i.v.	Concurrently to bleomycin challenge	Polarization of macrophages to M2 anti-inflammatory phenotype, ↓ Lung inflammation and fibrosis
Park et al., 2019	LPS or *E. coli*-induced ARDS	MVs from BM-MSCs, primed or not with Poly(I:C)	i.v.	1 h after injury	↑ Alveolar clearance, ↓ Bacterial load, protein permeability
Porzionato et al., 2019	Hyperoxia-induced bronchopulmonary dysplasia	EVs from UC-MSCs	i.t.	On days 3, 7, and 10 of hyperoxia	↓ Thickness of small pulmonary vessels
Silva et al., 2019	LPS-induced ARDS	EVs from 1 × 10^5^ BM-MSCs	i.v.	24 h after injury	EVs were less effective than parental MSCs at reducing lung injury, Priming with serum from injured mice did not enhance therapeutic responses
Varkouhi et al., 2019	*E. coli*-induced ARDS	MVs from 10 × 10^6^ UC-MSCs, primed or not with IFN-γ	i.v.	0.5 h after injury	↑ Bacterial phagocytosis and killing, ↓ Lung inflammation and injury Better effects were found in groups receiving IFN-γ-primed MSC-derived EVs vs. naive ones
Gao et al., 2020	PM2.5-induced ARDS	EVs from AD-MSCs	i.t.	1 h after injury	↓ Lung inflammation, apoptosis, pulmonary fibrosis (collagen fiber area and TGF-β levels) and ROS levels
Klinger et al., 2020	Sugen/hypoxia-induced pulmonary hypertension	EVs from human MSCs	i.v.	3 days after injury	Polarization of macrophages to M2 anti-inflammatory phenotype, ↓ Right ventricular pressure and hypertrophy
Wang et al., 2020	LPS-induced ARDS	EVs from 1 × 10^6^ AD-MSCs	i.v.	0.5 h after injury	miR-27a-3p transfer to macrophages Polarization of macrophages to M2 anti-inflammatory phenotype, ↓ Lung inflammation (neutrophil count inflammatory cytokine levels) and alveolar septum thickness
Yu et al., 2020	VILI	Exosomes from AD-MSCs	i.v.	1 h before injury	↓ Lung inflammation, alveolar-endothelial barrier hyperpermeability, ↑ expression of adherens junctions

*ARDS, acute respiratory distress syndrome; AD, adipose derived; BM, bone marrow; CLP, cecal ligation puncture; CM, conditioned medium; EV, extracelullar vesicle; HMGB, high mobility group box; IFN, interferon; IL, interleukin; i.n., intranasal; i.t., intratracheal; i.v., intravenous; KGF, keratinocyte growth factor; LPS, lipopolysaccharide; LTB, leukotriene; MIP, macrophage inflammatory protein; MRP, multidrug resistance protein; MSC, mesenchymsl stromal cell; MV, microvesicle; ROS, reactive oxygen species; TGF, transforming growth factor; TLR, Toll-like receptor; TSG, Tumor necrosis factor-inducible gene; TNF, tumor necrosis factor; UC, umbilical cord; VILI, ventilator-induced lung injury; WJ, Wharton’s Jelly.*

### Chronic Obstructive Pulmonary Disease

Chronic obstructive pulmonary disease is characterized by a restriction of airflow due to airway obstruction that is progressive and partially irreversible (Singh et al., 2019), among several other respiratory symptoms. During exacerbations, COPD patients often require pharmacological intervention and hospitalization to alleviate symptoms (Padilha et al., 2015; Global Initiative for Chronic Obstructive Lung Disease [GOLD], 2020).

Several studies have demonstrated that EVs can be used not only as a potential therapeutic strategy but also as an important biomarker of COPD diagnosis and prognosis. Some reports suggest that stress-induced lung-derived EVs participate in the pathogenetic cascade of COPD (Frydrychowicz et al., 2015; Fujita et al., 2018). For example, activated neutrophils are able to produce and secrete exosomes that carry surface-bound neutrophil elastase. This molecule can lead to extensive alveolar deterioration due to its ability to withstand degradation by α1-antitrypsin (Genschmer et al., 2019).

Infection is a major cause of complications in COPD patients, as it may result in exacerbations, which worsen the excessive production of mucus, prevent effective mucociliary clearance in the airways, and aggravate disease progression and severity (Sethi and Murphy, 2008). As virtually all cells are able to secrete EVs, analysis of these can help in the identification of specific features of the individual and pathogen, including the initial focus and etiologic agent of infection (Rodrigues et al., 2018). If pathogen-induced EVs are released during an infectious process, these can carry relevant molecular information regarding pathogen replication and interaction with host immune responses, as well as possible diagnostic bioindicators (Rodrigues et al., 2018). In this context, Takahashi et al. (2012) found that endothelial cell-derived EVs were overexpressed in COPD patients, especially during exacerbations, highlighting the potential of EVs to predict these exacerbations. Furthermore, studies observed that some miRNAs found within EVs may correlate directly with COPD severity by participating in disease development and progression (Savarimuthu Francis et al., 2014). Cigarette smoke exposure can modify the cargo of EVs released by BECs, with upregulation of several miRNAs (miR-500a-5p, miR-574-5p, miR-656-5p, miR-3180-5p, and miR-3913-5p) and downregulation of three miRNAs (miR-222-5p, miR-618, and miR-130b-5p) (Corsello et al., 2019). In another study reporting the effects of EV transfer on COPD pathogenesis, the authors indicated that microparticles can promote the release of a subset of miRNAs (miR-125a, miR-126, and miR-191) to resident macrophages, which are responsible for the removal of apoptotic cells (Serban et al., 2016). Exosomal miR-21 derived from BECs has been associated with myofibroblast differentiation in response to cigarette smoke, since smokers present a high level of exosomal miR-21 compared to healthy controls (Serban et al., 2016; Xu et al., 2018). Therefore, more studies investigating exosomal miRNA cargo should help further our understanding of the role of circulating miRNAs as potential biomarkers. Tan et al. (2017) evaluated the levels of serum-derived exosomes in COPD patients during exacerbations and in stable disease. These patients presented higher levels of serum-derived exosomes, which correlated with the levels of C-reactive protein (CRP), interleukin-6 (IL-6), and soluble tumor necrosis factor receptor 1 (sTNFR1) expression, suggesting a role of these vesicles during COPD exacerbations (Tan et al., 2017).

Some recent reports have indicated that most of the immunomodulatory actions and reparative ability of MSCs are recapitulated by their EVs and, therefore, MSC-derived EVs appear to be a viable substitute for whole cell-based therapy (Ahn et al., 2018; Khatri et al., 2018). In a model of elastase-induced emphysema, the effects of EVs harvested from adipose tissue-derived MSCs were compared with those of artificially developed nanovesicles obtained from this same cell type (Kim Y.S. et al., 2017). The authors found a higher proliferation rate of lung epithelial cells after exposure to lower doses of artificial nanovesicles than after exposure to the natural exosomes and even higher doses of adipose tissue-derived MSCs; this suggests that artificial nanovesicles might have both economic and clinical advantages in the treatment of individuals with emphysema (Kim Y.S. et al., 2017). Nevertheless, identification of the best route of administration for EV therapy in the clinics remains an important factor that must be investigated (Aliotta et al., 2016).

Some molecular engineering techniques have been investigated to modify and enhance the therapeutic potential of EVs. Both exogenous and endogenous modifications have been widely employed. The first consists of the incorporation of a desired cargo (RNAs, proteins, or even small-molecule compounds) into or onto isolated EVs, while the latter involves stimulating cells in a way that ensures the incorporation of the desired cargo into EVs during their synthesis (Wiklander et al., 2019). Co-incubation, electroporation, and sonication are some exogenous methods to package desired cargo into EVs that have demonstrated success in several studies. The incorporation of curcumin by EVs is a successful example that has been shown to improve the bioavailability of this compound and anti-inflammatory actions in distinct models of inflammation (Sun et al., 2010; Alvarez-Erviti et al., 2011; Didiot et al., 2016; Lamichhane et al., 2016). On the other hand, the most common endogenous methods include genetic modification of parent cells to induce overexpression of a certain RNA or protein, which in turn increases its level in EVs (Wiklander et al., 2019). EVs can serve as a “shield” and protect miRNAs from digestion and degradation, while also enabling an efficient delivery of the cargo to target cells (Etheridge et al., 2011; Minciacchi et al., 2015; Zhang et al., 2017). Furthermore, the use of patient-derived EVs can provide additional benefits, as these possess high cell specificity and may evade immune-system surveillance, thus lowering the risk of reaction or rejection in the recipient (Zhang et al., 2017).

### Pulmonary Infections

Lee et al. (2009) were able to demonstrate that certain bacterial species, such as *Bacillus subtilis* and *Staphylococcus aureus*, produce EVs. Kim et al. (2015) demonstrated that EVs derived from *Escherichia coli* cultures can increase IL-17A and TNF-α levels, thus intensifying lung inflammation in experimental emphysema. Furthermore, COPD patients possess lung-derived EV microbiomes that are significantly distinct from their whole lung tissue microbiomes (Kim H. J. et al., 2017).

In addition to bacterial infection, viruses are common triggers of COPD exacerbations (Hewitt et al., 2016). In fact, some reports suggest that respiratory viruses might have adapted to usurp resident cell-derived EVs for the transmission and dissemination of viral particles and virus-expressed molecules, such as miRNAs, mRNAs, and viral proteins (Altan-Bonnet, 2016). These can be then transported to uninfected neighbor cells or reach the bloodstream by distinct mechanisms (Rodrigues et al., 2018).

Conversely, EVs derived from swine bone marrow MSCs were able to reduce viral replication and shedding and secretion of pro-inflammatory mediators in a porcine model of influenza (Khatri et al., 2018). Based on the current knowledge of the therapeutic potential of MSC-derived EVs on lung diseases, a growing number of research groups has hypothesized that these bioactive products might be an effective strategy against severe acute respiratory syndrome coronavirus 2 (SARS-CoV-2), the novel coronavirus responsible for the ongoing active COVID-19 pandemic (Lopes-Pacheco et al., 2021). Due to the urgent need for an effective therapy against this devastating disease, administration of exosomes from allogeneic bone marrow MSCs (ExoFlo^TM^) was evaluated in a small, non-randomized, open-label cohort study of individuals with severe COVID-19. ExoFlo^TM^ demonstrated a good safety profile and was able to improve oxygenation and biomarkers associated with inflammation (Sengupta et al., 2020). This study had several limitations, such as the dearth of information regarding ExoFlo^TM^ (EV characterization, biological properties, proposed biological or therapeutic actions); efficacy remains to be demonstrated in large-scale clinical studies. A clinical trial evaluating exosome inhalation for SARS-CoV-2-induced pneumonia is in progress (NCT04491240).

The use of pathogen-derived EVs and/or their bioactive cargo as EV-based vaccines has also been investigated with some positive results (Roy et al., 2011). Clinical trials assessing EVs of viral origin demonstrated advantages compared to conventional vaccines against infectious diseases, since EVs cannot multiply or divide, which suggests that they are safer, less tumorigenic, and less infectious. Preclinical and clinical trials of EV-based vaccines used as antimicrobial treatments have observed good tolerability and feasibility. Nevertheless, further investigation is necessary to validate the use of EV-based vaccines in humans and demonstrate persistent immunostimulatory actions (Wiklander et al., 2019).

### Asthma

Asthma is a chronic inflammatory disease characterized by airway narrowing and hyperresponsiveness (GINA, 2020). The use of long-acting β_2_-adrenoceptor agonists combined with inhaled corticosteroids is able to achieve disease control in most asthmatic individuals; however, those with severe asthma might be resistant to treatment, and these drugs are unable to reverse established remodeling (Papierniak et al., 2013). Asthma presents distinct phenotypes, which have specific pathological and clinical features and respond differently to pharmacotherapies (Gauthier et al., 2015; Ray et al., 2015). These aspects are likely associated with individual genetic background, type of allergen, and which cell types participate in asthma pathogenesis (Stokes and Casale, 2016). In this context, different types of EVs, especially exosomes, have been recognized as important players in the inflammatory and remodeling processes of asthma.

Extracellular vesicles obtained from different sources may be used as disease markers or even have therapeutic potential in asthma. BALF-derived EVs from asthmatic patients demonstrated increased levels of IL-4 and leukotriene C4 (Torregrosa Paredes et al., 2012). In experimental allergic asthma, administration of BALF-derived EVs from asthmatic mice reduced airway inflammation and hyperresponsiveness (Prado et al., 2008). In another study, sensitization and challenge with house dust mite (HDM) extract promoted an 8.9-fold increase in EV content in BALF (Gon et al., 2017), which corroborates the observation that EV content can differ significantly among asthmatic patients (Francisco-Garcia et al., 2019). HDM exposure led to significant alterations in the expression of over 130 miRNAs in EVs, with an upregulation of 31 genes (including IL-5Ra and IL-13, which are potential targets of miR-574-5p and miR-346, respectively). The amount of lung cell-derived EVs was reduced when GW4869, an exosome generation inhibitor, was administered, ameliorating allergic asthma symptoms in a murine model (Gon et al., 2017).

In recent years, evidence has emerged regarding the therapeutic potential of MSC-derived EVs for the treatment of allergic asthma. MSC-derived EV therapy is able to upregulate IL-10 and transforming growth factor-β1 (TGF-β1), which in turn are responsible for the recruitment and expansion of regulatory T cells (Du et al., 2018), decrease the number of eosinophils in both lung tissue and BALF while also modulating airway remodeling (de Castro et al., 2017), and mitigate T_*H*_2/T_*H*_1-induced lung inflammation and airway hyperresponsiveness (Cruz et al., 2015). A recent clinical study demonstrated that administration of bone marrow-derived mononuclear cells was well-tolerated and safe and improved some quality of life parameters in three patients with severe asthma (Aguiar et al., 2020). Two clinical studies (NCT02192736 and NCT03137199) designed to evaluate the potential therapeutic actions of MSCs and their trophic factors in individuals with severe asthma are ongoing. However, the safety and efficacy of EVs in asthmatic patients remain to be investigated.

### Acute Respiratory Distress Syndrome

The acute respiratory distress syndrome is characterized by disruption of the alveolar-capillary membrane, with intense neutrophil inflow into the alveolar space, increased levels of pro-inflammatory cytokines/chemokines, surfactant dysfunction, and presence of protein-rich pulmonary edema fluid, resulting in severe hypoxemia (Lopes-Pacheco et al., 2020). Several therapeutic and supportive approaches have been developed; however, mortality rates remain high (35–45%) (Bellani et al., 2016).

Several cell therapies (MSCs, embryonic stem cells, induced pluripotent stem cells, endothelial progenitor cells) have been investigated as potential therapeutic strategies in experimental models of ARDS (Monsel et al., 2014; Lopes-Pacheco et al., 2020). The few early stage clinical trials that were completed showed a good safety profile of MSC administration (Zheng et al., 2014; McIntyre et al., 2018; Matthay et al., 2019), but efficacy still needs to be demonstrated in large-scale clinical studies.

In animal models of LPS-induced ARDS, intratracheal instillation of human MSC-derived MVs reduced lung edema (Zhu et al., 2014; Wang et al., 2020); however, knockdown of angiopoietin-1 or keratinocyte growth factor (KGF) mRNA in MSC-derived MVs abrogated these protective effects (Tang et al., 2017). Furthermore, administration of human MSC-derived MVs improved survival and decreased bacterial growth, protein permeability, and lung inflammation in experimental *E. coli*-induced pneumonia (Monsel et al., 2015). Recently, in an *ex vivo* model of severe bacterial pneumonia using perfused human lung tissue, intravenous instillation of MSC-derived EVs decreased bacterial load, lung edema, and protein permeability, restoring alveolar fluid clearance, corroborating previous findings in animal models (Park et al., 2019).

The reparative potential of MSC-derived EVs in lung endothelial and epithelial cells has been extensively investigated, as these are the main cell types injured in ARDS. Hu et al. (2018) observed that MSC-derived EVs were able to recover human lung vascular endothelial cells by transferring angiopoietin-1 mRNA to cells, preventing formation of actin “stress fibers.” However, exploring the actions of MSCs or their EVs on repair of pulmonary vascular dysfunction in ARDS will require future studies. Similarly, the effects of MSC-derived EVs on the bioenergetics of the alveolar epithelium have been explored in only a few investigations. Monsel et al. (2015) observed that MSC-derived EVs are able to restore intracellular ATP levels and survival of alveolar epithelial type II cells damaged by exposure to certain inflammatory factors [IL-1β, interferon-γ (IFN-γ), and TNF-α].

Among the mechanisms associated with the beneficial actions of MSC-derived EVs in ARDS is their ability to promote immunomodulatory responses, e.g., regulating T- and B-cells (Budoni et al., 2013; Chen et al., 2016; Di Trapani et al., 2016), and macrophage polarization, leading to a shift from the classical M1 to an anti-inflammatory M2-like profile (Ti et al., 2015; Abreu et al., 2018, 2019; Rodrigues et al., 2018; Wang et al., 2020) likely *via* transfer of miR-27a-3p (Wang et al., 2020). MSC-induced EVs can also increase the phagocytosis capacity of macrophages in part by mitochondrial transfer from EVs to damaged alveolar monocytes/macrophages, which results in enhanced oxidative phosphorylation (Morrison et al., 2017). The antimicrobial actions of EVs were even greater when MSCs were primed with a TLR-3 agonist or IFN-γ before vesicle production and release (Monsel et al., 2015; Park et al., 2019; Varkouhi et al., 2019). Conversely, priming MSCs with serum obtained from injured mice was not associated with enhancement of EV actions in LPS-induced ARDS (Silva et al., 2019). Antiviral effects of MSC-derived EVs have also been reported (Chan et al., 2016; Qian et al., 2016).

Acute respiratory distress syndrome patients who survive the initial inflammatory insult can progress to a dysregulated proliferative and fibrotic process in lung tissue (Burnham et al., 2014), thus impairing lung function and potentially compromising quality of life (Orme et al., 2003). In experimental ARDS, MSC-derived EVs reduced not only lung inflammation but also fibrosis (Gao et al., 2020). Although prevention of lung fibrosis following ARDS remains the best approach to improve long-term prognosis, a better understanding of the potential ability of MSC-derived EVs to prevent or reverse post-ARDS lung fibrosis is warranted.

### Idiopathic Pulmonary Fibrosis

Idiopathic pulmonary fibrosis represents ∼30% of all interstitial lung disorders and leads to respiratory failure within 5 years after disease onset (Sauleda et al., 2018). Tissue fibrosis is induced by elevated production of profibrotic mediators, mainly TGF-β. This growth factor has the ability to stimulate deposition of extracellular matrix components by promoting fibroblast activation/proliferation in damaged tissue, promoting persistence of apoptosis-resistant myofibroblasts, and activating the epithelial–mesenchymal transition (Lopes-Pacheco et al., 2016; de Oliveira et al., 2017). The potential of EVs in lung fibrosis remains to be further investigated.

Evidence of the role of exosomes in the pathophysiology of pulmonary fibrosis is still unclear. Some studies have shown that the profibrotic effects of TGF-β are regulated *via* programmed death ligand-1 (PD-L1). TGF-β induces PD-L1 *via* both Smad-dependent and -independent pathways; PD-L1 has also been shown to exert a previously unexpected role in the fibroproliferative actions of TGF-β and, when secreted into EVs, can act in a paracrine manner, providing greater opportunities for effective therapeutic intervention. A recent study demonstrated that TGF-β priming can considerably increase the amount of PD-L1 in fibroblast-derived EVs, which, in turn, induced a decrease in T-cell proliferation while increasing fibroblast migration in a PD-L1-dependent manner (Kang et al., 2019). Further investigations on the inhibition or promotion of profibrotic TGF-β signaling may reveal potential targets for the treatment of IPF and other lung fibrosis disorders.

Significant upregulation of miR-21-5p within serum-derived exosomes has been observed in experimental bleomycin-induced lung fibrosis (Makiguchi et al., 2016; Li et al., 2019). Baseline levels of miR-21-5p were found to correlate significantly with disease progression and were able to predict mortality during a 30-month follow-up in patients (Makiguchi et al., 2016; Li et al., 2019). MSC-derived EVs promoted immunomodulatory actions in experimental bleomycin-induced lung fibrosis, reducing both inflammation and remodeling (Tan et al., 2018; Mansouri et al., 2019). Another study also demonstrated the ability of MSC-derived EVs to suppress TGF-β-induced myofibroblast differentiation (Shentu et al., 2017).

It is important to note that quantification of the total amount of serum-derived exosomes can be largely variable. Therefore, implementation of standard methods to quantify certain microRNAs in clinical samples is still a challenge. The identification of more disease-specific biomarkers can be of great assistance to address this issue. As exosomes and microRNAs within cells might reflect pulmonary fibrosis status and severity, these may be combined with conventional biomarkers such as KL6, metalloprotease activity, and surfactant proteins A and D to obtain insight into the pathophysiology of IPF and patients’ clinical condition.

### Cystic Fibrosis

Cystic fibrosis is a genetic disorder caused by mutations in the gene that encodes the CF transmembrane conductance regulator (*CFTR*) protein, which plays an important role as a bicarbonate and chloride channel at the surface of epithelial cells. Although the disease leads to a multisystem dysfunction, the major causes of morbidity and mortality are related to the respiratory disorder (mucus accumulation in the airways, chronic inflammation, and persistent infections, which result in a progressive decline of lung function) (Lopes-Pacheco, 2016).

Although almost 2,100 *CFTR* gene variants have been identified, the most common CF-causing mutation is the deletion of a phenylalanine residue at position 508 (F508del), which is found in ∼80% of CF patients (Lopes-Pacheco, 2020). This mutation leads to misfolding of the CFTR protein, which is then prematurely degraded by proteasomes (Lopes-Pacheco, 2020). As a consequence, CF patients are more susceptible to infection by certain pathogens, including *S. aureus* and *Pseudomonas aeruginosa*. Additionally, high concentrations of IL-8 and other neutrophil chemokines are usually observed in CF airways, as is intense neutrophil recruitment (Downey et al., 2009). This chronic inflammatory process leads to increased levels of oxidative burst, elastase, and pro-inflammatory mediators in the airways (Tabary et al., 2006; Mitri et al., 2020).

Microvesicles derived from CF granulocytes have been associated with an extensive presence of neutrophils in the airways and aggregation of these structures on the epithelial surface of the respiratory tract in CF patients (Tabary et al., 2006; Asef et al., 2018). These vesicles are associated with chronic inflammation and with the pro-inflammatory response characteristic of CF. Furthermore, evidence suggests that CF epithelial cells release vesicles of a particular size into the airways and BALF, which can be useful as biomarkers (Baixauli et al., 2014). The amount and type of mucin on the surface of the exosome are also indicative of mucus obstruction in CF patients (Batson et al., 2016). Therefore, as CFTR function is impaired in CF epithelia, exosomes may be a useful tool to reestablish normal CFTR function (Vituret et al., 2016). Indeed, EVs have been investigated as vectors for the transfer of wild-type CFTR into CF cells (Vituret et al., 2016).

Based on the foregoing, EVs may be a promising therapeutic approach for CF, given their anti-inflammatory and antioxidant actions in experimental models. Although the precise EV cargo related to these actions remains to be further exploited, some authors reported a correlation between upregulation of peroxisome proliferator-activated receptor-γ (PPARγ) and EV administration, thus inducing a modulation of downstream effectors [NF-κB and heme oxygenase-1 (HO-1)] of these pathways (Zulueta et al., 2018). Nevertheless, most investigations have focused on the potential replacement of functional CFTR using exosomes as possible vectors. In this context, some reports demonstrated successful transduction of exosomes derived from A549 cells (a human adenocarcinoma alveolar basal epithelial cell line) and Calu-3 (an epithelial cell line) with an adenoviral vector overexpressing green fluorescent protein (GFP)-tagged CFTR. This approach was able to deliver the functional protein into CFTR-deficient epithelial cells (Vituret et al., 2016; Zulueta et al., 2018). Nevertheless, further studies are necessary to investigate the potential use of EVs as vectors in CF therapies.

### Extracellular Vesicle Therapy in Other Lung Diseases

Mesenchymal stromal cell-derived exosomes demonstrated cytoprotective actions similar to those of their parent cells in experimental pulmonary artery hypertension induced by hypoxia (Lee et al., 2012). MSC-derived EVs were also able to promote protective actions against the abnormal increase in right ventricular systolic pressure and right ventricular hypertrophy, which follow hypoxia exposure (Klinger et al., 2020). Specifically, MSC-derived exosome therapy upregulated hypoxia-induced mitogenic factors and suppressed inflammation by modulating early hypoxic signaling pathways. Furthermore, these vesicles were able to induce an alternative activation of alveolar macrophages (Klinger et al., 2020). Administration of either MSCs or their EVs was able to reduce mean pulmonary arterial pressure and mean right ventricular pressure in experimental monocrotaline-induced pulmonary arterial hypertension (Chen J. Y. et al., 2014; Aliotta et al., 2016). In another study, Willis et al. (2018) used a unique subset of MSC exosomes, namely, flotillin 1+ exosomes. The authors reported that administration of these vesicles led to improvements in lung development and morphology, as well as a reduction of tissue fibrosis and vascular endothelial remodeling in experimental hyperoxia in newborn mice (Willis et al., 2018). In another study using this model, umbilical cord MSC-derived EVs reduced lung inflammation and right ventricular hypertension in part due to exosomal TNF-stimulated gene 6 (TSG-6) (Chaubey et al., 2018).

Mesenchymal stromal cells-shed exosomes have demonstrated the ability to transfer certain regulatory microRNAs that resulted in modulation of TLR signaling and macrophage-secreted cytokine levels in an animal model of silica-induced lung inflammation and fibrosis (Phinney et al., 2015); miR-451, a factor that was highly abundant in these vesicles, is known to act as a macrophage migration inhibitory factor and inhibit TNF-induced signaling in response to silica (Phinney et al., 2015). In this context, Bandeira et al. (2019) demonstrated that MSC-derived EVs can reduce lung inflammation, fractional area of granuloma, and collagen deposition in experimental silicosis. Additionally, MSCs can transfer depolarized mitochondria *via* EVs that are captured by phagocytes, mainly macrophages. This process results in an enhancement of cellular bioenergetics (Morrison et al., 2017).

## Limitations and Outlook

Further research is required to better elucidate several questions and overcome barriers that currently preclude the use of EVs as a therapeutic approach in the clinic. First, the precise molecular cargo of EVs remains to be determined and may differ depending on factors such as cell type, culture conditions, and extraction and purification method, with potential impact on EV functional actions (Antounians et al., 2019). Some recent studies have identified certain microRNAs and proteins in EV cargo, which appear to be involved in EV therapeutic effects. These include miR-27a-3p, miR-30b-3p, miR-126, and miR-145 (Wu et al., 2018; Hao et al., 2019; Yi et al., 2019; Zhou et al., 2019; Wang et al., 2020) as well as angiopoietin-1, hepatocyte growth factor (HGF), and KGF (Zhu et al., 2014; Tang et al., 2017; Wang et al., 2017). Precise determination of EV cargo may help elucidate the mechanisms underlying EV-based therapies, as well as potentially enrich specific molecules to further enhance therapeutic actions.

Second, although some recommendations for EV isolation and characterization have been proposed, these procedures need to be further standardized. Most experimental studies have not included information regarding good manufacturing practices, which hinders investigation of the therapeutic actions of EVs in clinical studies. Several other factors need to be discussed and optimized to allow translation of EV research to clinical practice, including scalability, manufacturing, distribution, and regulation (for a review, see Maumus et al., 2020); establishment of quality control measures along each step of the manufacturing process may minimize variations, even among different batches. All of these technical hurdles have yet to be addressed, although some good manufacturing practices have been proposed elsewhere (Gimona et al., 2017; Witwer et al., 2019). Furthermore, EV isolation and loading also remain costly and inefficient processes.

Third, heterogeneity between studies and experimental designs should be considered; differences in models used, as well as genomics, gene regulation, expression and development, and epigenetic factors, must be taken into account before extrapolating findings to humans.

Fourth, EVs have been isolated from MSCs derived from different sources. The most commonly used MSC sources have been bone marrow, adipose tissue, and umbilical cord, which are known to exert distinct degrees of efficacy when living cells are administered therapeutically (Antunes et al., 2014; Silva et al., 2018; Kern et al., 2019). The best dose and route of administration of EVs to achieve the greatest therapeutic response in each lung disease remain to be determined (Cruz and Rocco, 2020; Lopes-Pacheco et al., 2020). In fact, the dose and route of administration of EVs have been largely variable across studies, which poses a substantial challenge to comparative analysis among these investigations. The therapeutic index and biodistribution of EVs remain unknown. In this context, a single dose of EVs has been demonstrated to induce therapeutic effects in several studies, but multiple doses should be considered, as this approach has consistently been associated with superior outcomes in cell-based therapy (Lopes-Pacheco et al., 2013; Poggio et al., 2018; Castro et al., 2020). Lastly, most *in vivo* studies have been performed in small animals (mainly rodents), with only a few reports in large animal models or *ex vivo* human lungs.

In conclusion, EV-based therapy has demonstrated the ability to reduce lung inflammation and remodeling in several animal models and holds therapeutic promise for clinical use in lung diseases. However, despite the effects observed in experimental models, additional studies are required to develop an effective EV-based lung therapy to be used in the clinical scenario.

## Author Contributions

SA and ML-P contributed to the design and conceptualization, original draft, editing, and review for intellectual content. DW contributed to the review for intellectual content. PR contributed to the design and conceptualization, editing, and review for intellectual content. All the authors read and approved the final version of the manuscript.

## Conflict of Interest

The authors declare that the research was conducted in the absence of any commercial or financial relationships that could be construed as a potential conflict of interest.

## Footnotes

1 http://www.microvesicles.org

2 http://www.exocarta.org

## References

[B1] Abreu S. C. Lopes-Pacheco M. da Silva A. L. Xisto D. G. de Oliveira T. B. Kitoko J. Z. (2018). Eicosapentaenoic acid enhances the effects of mesenchymal stromal cell therapy in experimental allergic asthma. *Front. Immunol.* 9:1147. 10.3389/fimmu.2018.01147 29881388 PMC5976792

[B2] Abreu S. C. Xisto D. G. de Oliveira T. B. Blanco N. G. de Castro L. L. Kitoko J. Z. (2019). Serum from asthmatic mice potentiates the therapeutic effects of mesenchymal stromal cells in experimental allergic asthma. *Stem Cells Transl. Med.* 8 301–312. 10.1002/sctm.18-0056 30426724 PMC6392406

[B3] Aguiar F. S. Melo A. S. Araújo M. A. S. Cardoso A. P. de Souza S. A. L. Lopes-Pacheco M. (2020). Autologous bone marrow-derived mononuclear cell therapy in three patients with severe asthma. *Stem Cell Res. Ther.* 11:167. 10.1186/s13287-020-01675-x PMC7193384 32357905

[B4] Ahn S. Y. Park W. S. Kim Y. E. Sung D. K. Sung S. I. Ahn J. Y. (2018). Vascular endothelial growth factor mediates the therapeutic efficacy of mesenchymal stem cell-derived extracellular vesicles against neonatal hyperoxic lung injury. *Exp. Mol. Med.* 50:26. 10.1038/s12276-018-0055-8 PMC5938045 29650962

[B5] Akyurekli C. Le Y. Richardson R. B. Fergusson D. Tay J. Allan D. S. (2015). A systematic review of preclinical studies on the therapeutic potential of mesenchymal stromal cell-derived microvesicles. *Stem Cell Rev. Rep.* 11 150–160. 10.1007/s12015-014-9545-9 25091427

[B6] Aliotta J. M. Pereira M. Wen S. Dooner M. S. Del Tatto M. Papa E. (2016). Exosomes induce and reverse monocrotaline-induced pulmonary hypertension in mice. *Cardiovasc. Res.* 110 319–330. 10.1093/cvr/cvw054 26980205 PMC4872877

[B7] Almqvist N. Lonnqvist A. Hultkrantz S. Telemo E. (2008). Serum-derived exosomes from antigen-fed mice prevent allergic sensitization in a model of allergic asthma. *Immunology* 125 21–27. 10.1111/j.1365-2567.2008.02812.x 18355242 PMC2526256

[B8] Altan-Bonnet N. (2016). Extracellular vesicles are the Trojan horses of viral infection. *Curr. Opin. Microbiol.* 32 77–81. 10.1016/j.mib.2016.05.004 27232382 PMC4983493

[B9] Alvarez-Erviti L. Seow Y. Yin H. Betts C. Lakhal S. Wood M. J. A. (2011). Delivery of siRNA to the mouse brain by systemic injection of targeted exosomes. *Nat. Biotechnol.* 29 341–345. 10.1038/nbt.1807 21423189

[B10] Antounians L. Tzanetakis A. Pellerito O. Catania V. D. Sulistyo A. Montalva L. (2019). The regenerative potential of amniotic fluid stem cells extracellular vesicles: lessons learned by comparing different isolation techniques. *Sci. Rep.* 9:1837. 10.1038/s41598-018-38320-w PMC6372651 30755672

[B11] Antunes M. A. Abreu S. C. Cruz F. F. Teixeira A. C. Lopes-Pacheco M. Bandeira E. (2014). Effects of different mesenchymal stromal cell sources and delivery routes in experimental emphysema. *Respir. Res.* 15:118. 10.1186/s12931-014-0118-x PMC4189723 25272959

[B12] Asef A. Mortaz E. Jamaati H. Velayati A. (2018). Immunologic role of extracellular vesicles and exosomes in the pathogenesis of cystic fibrosis. *Tanaffos* 17 66–72. 30627176 PMC6320567

[B13] Atkin-Smith G. K. Tixeira R. Paone S. Mathivanan S. Colling C. Liem M. (2015). A novel mechanism of generating extracellular vesicles during apoptosis via a beads-on-a-string membrane structure. *Nat. Commun.* 6:7439. 10.1038/ncomms8439 PMC4490561 26074490

[B14] Baglio S. R. Pegtel D. M. Baldini N. (2012). Mesenchymal stem cell secreted vesicles provide novel opportunities in (stem) cell-free therapy. *Front. Physiol*. 3:359. 10.3389/fphys.2012.00359 22973239 PMC3434369

[B15] Baixauli F. López-Otín C. Mittelbrunn M. (2014). Exosomes and autophagy: coordinated mechanisms for the maintenance of cellular fitness. *Front. Immunol.* 5:403. 10.3389/fimmu.2014.00403 25191326 PMC4138502

[B16] Bandeira E. Oliveira H. Silva J. D. Menna-Barreto R. F. S. Takyia C. M. Suk J. S. (2019). Therapeutic effects of adipose-tissue-derived mesenchymal stromal cells and their extracellular vesicles in experimental silicosis. *Respir. Res.* 19:104. 10.1186/s12931-018-0802-3 PMC5975461 29843724

[B17] Bari E. Ferrarotti I. Di Silvestre D. Grisoli P. Barzon V. Balderacchi A. (2019). Adipose mesenchymal extracellular vesicles as alpha-1-antitrypsin physiological delivery systems for lung regeneration. *Cells* 8:E965. 10.3390/cells8090965 31450843 PMC6770759

[B18] Batson B. Arifuzzaman M. Haridass P. Dand H. Mieczkowski P. Ribeiro C. (2016). Qualitative and quantitative changes of gel forming mucins and exosomes in response to infection and inflammation in the airways. *Am. J. Respir. Crit. Care Med.* 2016 A5565–A5565.

[B19] Bell G. I. Meschino M. T. Hughes-Large J. M. Broughton H. C. Xenocostas A. Hess D. A. (2012). Combinatorial human progenitor cell transplantation optimizes islet regeneration through secretion of paracrine factors. *Stem Cells Dev.* 21 1863–1876. 10.1089/scd.2011.0634 22309189

[B20] Bellani G. Laffey J. G. Pham T. Fan E. Brochard L. Esteban A. (2016). Epidemiology, patterns of care, and mortality for patients with acute respiratory distress syndrome in intensive care units in 50 countries. *JAMA* 315 788–800. 10.1001/jama.2016.0291 26903337

[B21] Bourdonnay E. Zaslona Z. Penke L. R. K. Speth J. M. Schneider D. J. Przybranowski S. (2015). Transcellular delivery of vesicular SOCS proteins from macrophages to epithelial cells blunts inflammatory signaling. *J. Exp. Med.* 212 729–742. 10.1084/jem.20141675 25847945 PMC4419346

[B22] Braun R. K. Chetty C. Balasubramaniam V. Centanni R. Haraldsdottir K. Hematti P. (2018). Intraperitoneal injection of MSC-derived exosomes prevent experimental bronchopulmonary dysplasia. *Biochem. Biophys. Res. Commun.* 503 2653–2658. 10.1016/j.bbrc.2018.08.019 30093115 PMC6398932

[B23] Bruno S. Camussi G. (2013). Role of mesenchymal stem cell-derived microvesicles in tissue repair. *Pediatr. Nephrol.* 28 2249–2254. 10.1007/s00467-013-2413-z 23386109

[B24] Bruno S. Deregibus M. C. Camussi G. (2015). The secretome of mesenchymal stromal cells: role of extracellular vesicles in immunomodulation. *Immunol. Lett.* 168 154–158. 10.1016/j.imlet.2015.06.007 26086886

[B25] Budoni M. Fierabracci A. Luciano R. Petrini S. Di Ciommo V. Muraca M. (2013). The immunosuppressive effect of mesenchymal stromal cells on B lymphocytes is mediated by membrane vesicles. *Cell Transpl.* 22 369–379. 10.3727/096368911x582769b 23433427

[B26] Burnham E. L. Janssen W. J. Riches D. W. Moss M. Downey G. P. (2014). The fibroproliferative response in acute respiratory distress syndrome: mechanisms and clinical significance. *Eur. Respir. J.* 43 276–285. 10.1183/09031936.00196412 23520315 PMC4015132

[B27] Camussi G. Deregibus M. C. Bruno S. Biancone L. (2010). Exosomes/microvesicles as a mechanism of cell-to-cell communication. *Kidney Int.* 78 838–848. 10.1038/ki.2010.278 20703216

[B28] Camussi G. Deregibus M. C. Cantaluppi V. (2013). Role of stem-cell-derived microvesicles in the paracrine action of stem cells. *Biochem. Soc. Trans.* 41 283–287. 10.1042/bst20120192 23356298

[B29] Castro L. L. Kitoko J. Z. Xisto D. G. Olsen P. C. Guedes H. L. M. (2020). Multiple doses of adipose tissue-derived mesenchymal stromal cells induce immunosuppression in experimental asthma. *Stem Cells Transl. Med.* 9 250–260. 10.1002/sctm.19-0120 31746562 PMC6988761

[B30] Chan M. C. Kuok D. I. Leung C. Y. Hui K. P. Y. Valkenburg S. A. Lau E. H. Y. (2016). Human mesenchymal stromal cells reduce influenza A H5N1-associated acute lung injury in vitro and in vivo. *Proc. Natl. Acad. Sci. U.S.A.* 113 3621–3626. 10.1073/pnas.1601911113 26976597 PMC4822574

[B31] Chaubey S. Thueson S. Ponnalagu D. Alam M. A. Gheorghe C. P. Aghai Z. (2018). Early gestational mesenchymal stem cell secretome attenuates experimental bronchopulmonary dysplasia in part via exosome-associated factor TSG-6. *Stem Cell Res. Ther.* 9:173. 10.1186/s13287-018-0903-4 PMC6019224 29941022

[B32] Chen J. Liu Z. Hong M. M. Zhang H. Chan C. Xiao M. (2014). Proangiogenic compositions of microvesicles derived from human umbilical cord mesenchymal stem cells. *PLoS One* 9:e115316. 10.1371/journal.pone.0115316 25514634 PMC4267846

[B33] Chen J. Y. An R. Liu Z. J. Wang J.-J. Chen S.-Z. Hong M.-M. (2014). Therapeutic effects of mesenchymal stem cell-derived microvesicles on pulmonary arterial hypertension in rats. *Acta Pharmacol. Sin.* 35 1121–1128. 10.1038/aps.2014.61 25088001 PMC4155533

[B34] Chen W. Huang Y. Han J. Yu L. Li Y. Lu Z. (2016). Immunomodulatory effects of mesenchymal stromal cells-derived exosome. *Immunol. Res.* 64 831–840. 10.1007/s12026-016-8798-6 27115513

[B35] Chen W.-X. Zhou J. Zhou S.-S. Zhang Y.-D. Ji T.-Y. Zhang X.-L. (2020). Microvesicles derived from human Wharton’s jelly mesenchymal stem cells enhance autophagy and ameliorate acute lung injury via delivery of miR-100. *Stem Cell Res. Ther.* 11:113. 10.1186/s13287-020-01617-7 PMC7071666 32169098

[B36] Cocucci E. Racchetti G. Meldolesi J. (2009). Shedding microvesicles: artefacts no more. *Trends Cell Biol.* 19 43–51. 10.1016/j.tcb.2008.11.003 19144520

[B37] Collino F. Deregibus M. C. Bruno S. Sterpone L. Aghmo G. Viltono L. (2010). Microvesicles derived from adult human bone marrow and tissue specific mesenchymal stem cells shuttle selected pattern of miRNAs. *PLoS One* 5:e11803. 10.1371/journal.pone.0011803 20668554 PMC2910725

[B38] Cordazzo C. Petrini S. Neri T. Lombardi S. Carmazzi Y. Pedrinelli R. (2014). Rapid shedding of proinflammatory microparticles by human mononuclear cells exposed to cigarette smoke is dependent on Ca^2+^ mobilization. *Inflamm. Res.* 63 539–547. 10.1007/s00011-014-0723-7 24599284

[B39] Corsello T. Kudlicki A. S. Garofalo R. P. Casola A. (2019). Cigarette smoke condensate exposure changes RNA content of extracellular vesicles released from small airway epithelial cells. *Cells* 8:1652. 10.3390/cells8121652 31861112 PMC6953119

[B40] Cruz F. F. Borg Z. D. Goodwin M. Sokocevic D. Wagner D. E. Coffey A. (2015). Systemic administration of human bone marrow-derived mesenchymal stromal cell extracellular vesicles ameliorates Aspergillus Hyphal extract-induced allergic airway inflammation in immunocompetent mice. *Stem Cells Transl. Med.* 4 1302–1316. 10.5966/sctm.2014-0280 26378259 PMC4622402

[B41] Cruz F. F. Rocco P. R. M. (2020). The potential of mesenchymal stem cell therapy for chronic lung disease. *Expert Rev. Respir. Med.* 14 31–39. 10.1080/17476348.2020.1679628 31608724

[B42] de Castro L. L. Lopes-Pacheco M. Weiss D. J. Cruz F. F. Rocco P. R. M. (2019). Current understanding of the immunosuppressive properties of mesenchymal stromal cells. *J. Mol. Med.* 97 605–618. 10.1007/s00109-019-01776-y 30903229

[B43] de Castro L. L. Xisto D. G. Kitoko J. Z. Cruz F. F. Olsen P. C. Redondo P. A. G. (2017). Human adipose tissue mesenchymal stromal cells and their extracellular vesicles act differentially on lung mechanics and inflammation in experimental allergic asthma. *Stem Cell Res. Ther.* 8:151. 10.1186/s13287-017-0600-8 PMC5482954 28646903

[B44] de Oliveira H. D. de Melo E. B. B. Silva J. D. Kitoko J. Z. Gutfilen B. Barboza T. (2017). Therapeutic effects of boné marrow-derived mononuclear cells from healthy or silicotic donors on recipient silicosis mice. *Stem Cell Res. Ther.* 8:259. 10.1186/s13287-017-0699-7 PMC5681761 29126438

[B45] Deregibus M. C. Figliolini F. D’Antico S. Manzini P. M. Pasquino C. De Lena M. (2016). Charge-based precipitation of extracellular vesicles. *Int. J. Mol. Med.* 38 1359–1366. 28025988 10.3892/ijmm.2016.2759 PMC5065305

[B46] Di Trapani M. Bassi G. Midolo M. Gatti A. Kamga P. T. Cassaro A. (2016). Differential and transferable modulatory effects of mesenchymal stromal cell-derived extracellular vesicles on T. B and NK cell functions. *Sci. Rep.* 6:24120. 10.1038/srep24120 PMC4829861 27071676

[B47] Didiot M. C. Hall L. M. Coles A. H. Haraszti R. A. Godinho B. M. Chase K. (2016). Exosome-mediated delivery of hydrophobically modified siRNA for huntingtin mRNA silencing. *Mol. Ther.* 24 1836–1847. 10.1038/mt.2016.126 27506293 PMC5112038

[B48] Dooner M. S. Aliotta J. M. Pimentel J. Dooner G. J. Abedi M. Colvin G. (2008). Conversion potential of marrow cells into lung cells fluctuates with cytokine-induced cell cycle. *Stem Cells Dev.* 17 207–219. 10.1089/scd.2007.0195 18447637

[B49] Downey D. G. Bell S. C. Elborn J. S. (2009). Neutrophils in cystic fibrosis. *Thorax* 64 81–88. 19103874 10.1136/thx.2007.082388

[B50] Du Y. M. Zhuansun Y. X. Chen R. Lin L. Lin Y. Li J.-G. (2018). Mesenchymal stem cell exosomes promote immunosuppression of regulatory T cells in asthma. *Exp. Cell. Res.* 363 114–120. 10.1016/j.yexcr.2017.12.021 29277503

[B51] Eirin A. Riester S. M. Zhu X. Y. Tang H. Evans J. M. O’Brien D. (2014). MicroRNA and mRNA cargo of extracellular vesicles from porcine adipose tissue-derived mesenchymal stem cells. *Gene* 551 55–64. 10.1016/j.gene.2014.08.041 25158130 PMC4174680

[B52] Eissa N. T. (2013). The exosome in lung diseases: message in a bottle. *J. Allergy Clin. Immunol.* 131 904–905. 10.1016/j.jaci.2013.01.021 23360758

[B53] Etheridge A. Lee I. Hood L. Galas D. Wang K. (2011). Extracellular microRNA: a new source of bioindicators. *Mutat. Res.* 717 85–90. 21402084 10.1016/j.mrfmmm.2011.03.004 PMC3199035

[B54] Fang S.-B. Zhang H.-Y. Wang C. He B.-X. Liu X.-Q. Meng X.-C. (2020). Small extracellular vesicles derived from human mesenchymal stromal cells prevent group 2 innate lymphoid cell-dominant allergic airway inflammation through delivery of miR-146a-5p. *J. Extracell. Ves.* 9:1723260. 10.1080/20013078.2020.1723260 32128074 PMC7034457

[B55] Francisco-Garcia A. S. Garrido-Martín E. M. Rupani H. Lau L. C. K. Martinez-Nunez R. T. Howarth P. H. (2019). Small RNA species and microRNA Profiles are altered in severe asthma nanovesicles from broncho alveolar lavage and associate with impaired lung function and inflammation. *Noncod. RNA* 5:51. 10.3390/ncrna5040051 31684064 PMC6958500

[B56] Frydrychowicz M. Kolecka-Bednarczyk A. Madejczyk M. Yasar S. Dworachi G. (2015). Exosomes - structure, biogenesis and biological role in non-small-cell lung cancer. *Scand. J. Immunol.* 81 2–10. 10.1111/sji.12247 25359529

[B57] Fujita Y. Kadota T. Araya J. Ochiya T. Kuwano K. (2018). Extracellular vesicles: new players in lung immunity. *Am. J. Respir. Cell Mol. Biol.* 58 560–565. 29115853 10.1165/rcmb.2017-0293TR

[B58] Fujita Y. Kosaka N. Araya J. Kuwano K. Ochiya T. (2015). Extracellular vesicles in lung microenvironment and pathogenesis. *Trends Mol. Med.* 21 533–542. 10.1016/j.molmed.2015.07.004 26231094

[B59] Galleu A. Riffo-Vasquez Y. Trento C. Lomas C. Dolvetti L. Cheung T. S. (2017). Apoptosis in mesenchymal stromal cells induces in vivo recipiente-mediated immunomodulation. *Sci. Transl. Med.* 9:eaam7828. 10.1126/scitranslmed.aam7828 29141887

[B60] Gámez-Valero A. Monguió-Tortajada M. Carreras-Planella L. La Franquesa M. Beyer K. Borràs F. E. (2016). Size-exclusion chromatography-based isolation minimally alters extracellular vesicles’ characteristics compared to precipitating agents. *Sci. Rep.* 6:33641. 10.1038/srep33641 PMC5027519 27640641

[B61] Gao Y. Sun J. Dong C. Zhao M. Hu Y. Jin F. (2020). Extracellular vesicles derived from adipose mesenchymal stem cells alleviate PM2.5-induced lung injury and pulmonary fibrosis. *Med. Sci. Monit.* 26:e922782. 10.12659/MSM.922782 PMC7191958 32304204

[B62] Gauthier M. Ray A. Wenzel S. E. (2015). Evolving concepts of asthma. *Am. J. Respir. Crit. Care Med.* 192 660–668. 10.1164/rccm.201504-0763pp 26161792 PMC5447293

[B63] Genschmer K. R. Russell D. W. Lal C. Szul T. Bratcher P. E. Noerager B. D. (2019). Activated PMN exosomes: pathogenic entities causing matrix destruction and disease in the lung. *Cell* 176 113–126. 10.1016/j.cell.2018.12.002 30633902 PMC6368091

[B64] Giebel B. Lambros K. Verena B. (2017). Clinical potential of mesenchymal stem/stromal cell-derived extracellular vesicles. *Stem Cell Invest.* 4:84. 10.21037/sci.2017.09.06 29167805 PMC5676188

[B65] Gimona M. Pachler K. Laner-Plamberger S. Schallmoser K. Rohde E. (2017). Manufacturing of human extracellular vesicle-based therapeutics for clinical use. *Int. J. Mol. Sci.* 18:1190. 10.3390/ijms18061190 28587212 PMC5486013

[B66] Global Initiative for Chronic Obstructive Lung Disease [GOLD] (2020). *Global Strategy for the Diagnosis, Management, and Prevention of Chronic Obstructive Pulmonary Disease.* Available online at: https://goldcopd.org/gold-reports/ (accessed August 30, 2020).

[B67] GINA (2020). *Global Strategy for Asthma Management and Prevention. Global Initiative for Asthma 2020.* Available online at: https://ginasthma.org/2020-gina-report-global-strategy-for-asthma- management-and-prevention/ (accessed August 30, 2020).

[B68] Gomzikova M. O. James V. Rizvanov A. A. (2019). Therapeutic application of mesenchymal stem cells derived extracellular vesicles for immunomodulation. *Front. Immunol.* 10:2663. 10.3389/fimmu.2019.02663 31849929 PMC6889906

[B69] Gon Y. Maruoka S. Inoue T. Kuroda K. Yamagishi K. Kozu Y. (2017). Selective release of miRNAs via extracellular vesicles is associated with house-dust mite allergen-induced airway inflammation. *Clin. Exp. Allergy* 47 1586–1598. 10.1111/cea.13016 28859242

[B70] Gon Y. Shimizu T. Mizumura K. Maruoka S. Hikichi M. (2020). Molecular techniques for respiratory diseases: MicroRNA and extracellular vesicles. *Respirology* 25 149–160. 10.1111/resp.13756 31872560

[B71] Goolaerts A. Pellan-Randrianarison N. Larghero J. Vanneaus V. Uzunhan Y. Gille T. (2014). Conditioned media from mesenchymal stromal cells restore sodium transport and preserve epithelial permeability in an in vitro model of acute alveolar injury. *Am. J. Physiol. Lung Cell Mol. Physiol.* 306 L975–L985. 24682451 10.1152/ajplung.00242.2013 PMC4042188

[B72] Gowen A. Shahjin F. Chand S. Odegaard K. E. Yelamanchili S. V. (2020). Mesenchymal stem cell-derived extracellular vesicles: challenges in clinical applications. *Front. Cell Dev. Biol.* 8:149. 10.3389/fcell.2020.00149 32226787 PMC7080981

[B73] Guiot J. Struman I. Louis E. Louis R. Malaise M. Njock M.-S. (2019). Exosomal miRNAs in lung diseases: from biologic function to therapeutic targets. *J. Clin. Med.* 8:1345. 10.3390/jcm8091345 31470655 PMC6781233

[B74] Hao Q. Gudapati V. Monsel A. Park J. H. Ho S. (2019). Mesenchymal stem cell-derived extracellular vesciles decrese lung injury in Mice. *J. Immunol.* 203 1961–1972. 31451675 10.4049/jimmunol.1801534 PMC6760999

[B75] Harding C. Heuser J. Stahl P. (1983). Receptor-mediated endocytosis of transferrin and recycling of the transferrin receptor in rat reticulocytes. *J. Cell. Biol.* 97 329–339. 10.1083/jcb.97.2.329 6309857 PMC2112509

[B76] Hayes M. Curley G. F. Masterson C. Devaney J. O’Toole D. Laffey J. G. (2015). Mesenchymal stromal cells are more effective than the MSC secretome in diminishing injury and enhancing recovery following ventilator-induced lung injury. *Intens. Care Med. Exp.* 3:29. 10.1186/s40635-015-0065-y PMC4607685 26472334

[B77] Henne W. M. Stenmark H. Emr S. D. (2013). Molecular mechanisms of the membrane sculpting ESCRT pathway. *Cold Spring Harb. Perspect. Biol.* 5:a016766. 10.1101/cshperspect.a016766 24003212 PMC3753708

[B78] Hessvik N. P. Llorente A. (2018). Current knowledge on exosome biogenesis and release. *Cell. Mol. Life Sci.* 75 193–208. 10.1007/s00018-017-2595-9 28733901 PMC5756260

[B79] Hewitt R. Farne H. Ritchie A. Luke E. Johnston S. L. Mallia P. (2016). The role of viral infections in exacerbations of chronic obstructive pulmonary disease and asthma. *Ther. Adv. Respir. Dis.* 10 158–174. 26611907 10.1177/1753465815618113 PMC5933560

[B80] Hofmann N. A. Ortner A. Jacamo R. O. Reinisch A. Schallmoser K. Rohban R. (2012). Oxygen sensing mesenchymal progenitors promote neo-vasculogenesis in a humanized mouse model in vivo. *PLoS One* 7:e44468. 10.1371/journal.pone.0044468 22970226 PMC3436890

[B81] Hu S. Park J. Liu A. Lee J. Zhang X. Hao Q. (2018). Mesenchymal stem cell microvesicles restore protein permeability across primary cultures of injured human lung microvascular endothelial cells. *Stem Cells Transl. Med.* 7 615–624. 10.1002/sctm.17-0278 29737632 PMC6090509

[B82] Ismail N. Wang Y. Dakhlallah D. Moldovan L. Agarwal K. Batter K. (2013). Macrophage microvesicles induce macrophage differentiation and miR-223 transfer. *Blood* 121 984–995. 10.1182/blood-2011-08-374793 23144169 PMC3567345

[B83] Kadota T. Fujita Y. Yoshioka Y. Araya J. Kuwano K. Ochiya T. (2016). Extracellular vesicles in chronic obstructive pulmonary disease. *Int. J. Mol. Sci.* 17:1801. 10.3390/ijms17111801 PMC5133802 27801806

[B84] Kang J.-H. Jung M.-Y. Choudhury M. Leof E. B. (2019). Transforming growth factor beta induces fibroblasts to express and release the immunomodulatory protein PD-L1 into extracellular vesicles. *FASEB J.* 34 2213–2226. 10.1096/fj.201902354r 31907984

[B85] Katsuda T. Kosaka N. Takeshita F. Ochiya T. (2013). The therapeutic potential of mesenchymal stem cell-derived extracellular vesicles. *Proteomics* 13 1637–1653. 23335344 10.1002/pmic.201200373

[B86] Kern S. Eichler H. Stoeve J. Klüter H. Bieback K. (2019). Comparative analysis of mesenchymal stem cells from bone marrow, umbilical cord blood, or adipose tissue. *Stem Cells* 24 1294–1301. 10.1634/stemcells.2005-0342 16410387

[B87] Kerr J. F. Wyllie A. H. Currie A. R. (1972). Apoptosis: a basic biological phenomenon with wide-ranging implications in tissue kinetics. *Br. J. Cancer* 26 239–257. 10.1038/bjc.1972.33 4561027 PMC2008650

[B88] Kesimer M. Gupta R. (2015). Physical characterization and profiling of airway epithelial derived exosomes using light scattering. *Methods* 87 59–63. 10.1016/j.ymeth.2015.03.013 25823850 PMC4584172

[B89] Kesimer M. Scull M. Brighton B. DeMaria G. Burns K. O’Neal W. (2009). Characterization of exosome-like vesicles released from human tracheobronchial ciliated epithelium: a possible role in innate defense. *FASEB J.* 23 1858–1868. 10.1096/fj.08-119131 19190083 PMC2698655

[B90] Khatri M. Richardson L. A. Meulia T. (2018). Mesenchymal stem cell-derived extracellular vesicles attenuate influenza virus-induced acute lung injury in a pig model. *Stem Cell Res. Ther.* 9:17. 10.1186/s13287-018-0774-8 PMC5789598 29378639

[B91] Kim H. J. Kim Y. S. Kim K. H. Choi J.-P. Kim Y.-K. Yun S. (2017). The microbiome of the lung and its extracellular vesicles in nonsmokers, healthy smokers and COPD patients. *Exp. Mol. Med.* 49:e316. 10.1038/emm.2017.7 28408748 PMC5420800

[B92] Kim Y. S. Kim J. Y. Cho R. Shin D.-M. Lee S. W. Oh Y.-M. (2017). Adipose stem cell-derived nanovesicles inhibit emphysema primarily via an FGF2-dependent pathway. *Exp. Mol. Med.* 49:e284. 10.1038/emm.2016.127 28082743 PMC5291836

[B93] Kim Y. S. Lee W.-H. Choi E.-J. Choi J.-P. Heo Y. J. Gho Y. S. (2015). Extracellular vesicles derived from Gram-negative bacteria, such as *Escherichia coli*, induce emphysema mainly via IL-17A-mediated neutrophilic inflammation. *J. Immunol.* 194 3361–3368. 10.4049/jimmunol.1402268 25716999

[B94] Kinoshita T. Yip K. W. Spence T. Liu F.-F. (2017). MicroRNAs in extracellular vesicles: potential cancer bioindicators. *J. Hum. Genet.* 62 67–74. 10.1038/jhg.2016.87 27383658

[B95] Klinger J. R. Pereira M. Del Tatto M. Brodsky A. S. Wu Q. K. Dooner M. S. (2020). Mesenchymal stem cells extracellular vesicles reverse Sugen/hypoxia pulmonary hypertension in rats. *Am. J. Respir. Cell. Mol. Biol.* 62 577–587. 10.1165/rcmb.2019-0154oc 31721618 PMC7193796

[B96] Konoshenko M. Y. Lekchnov E. A. Vlassov A. V. Laktionov P. P. (2018). Isolation of extracellular vesicles: general methodologies and latest trends. *Biomed. Res. Int.* 2018:8545347. 10.1155/2018/8545347 PMC5831698 29662902

[B97] Kowal J. Tkach M. Theìry C. (2014). Biogenesis and secretion of exosomes. *Curr. Opin. Cell Biol.* 29 116–125. 10.1016/j.ceb.2014.05.004 24959705

[B98] Kulshreshtha A. Ahmad T. Agrawal A. Ghosh B. (2013). Proinflammatory role of epithelial cell-derived exosomes in allergic airway inflammation. *J. Allergy Clin. Immunol.* 131 1194–1203. 10.1016/j.jaci.2012.12.1565 23414598

[B99] Lacy S. H. Woeller C. F. Thatcher T. H. Pollack S. J. Small E. M. Sime P. J. (2019). Activated human lung fibroblasts produce extracellular vesicles with antifibrotic prostaglandins. *Am. J. Respir. Cell Mol. Biol.* 60 269–278. 10.1165/rcmb.2017-0248oc 30265126 PMC6397975

[B100] Lai R. C. Yeo R. W. Lim S. K. (2015). Mesenchymal stem cell exosomes. *Semin. Cell Dev. Biol.* 40 82–88. 25765629 10.1016/j.semcdb.2015.03.001

[B101] Lalu M. M. McIntyre L. Pugliese C. Fergusson D. Winston B. W. Marshall J. C. (2012). Safety of cell therapy with mesenchymal stromal cells (SafeCell), a systematic review and meta-analysis of clinical trials. *PLoS One* 7:e47559. 10.1371/journal.pone.0047559 23133515 PMC3485008

[B102] Lamichhane T. N. Jeyaram A. Patel D. B. Parajuli B. Livingston N. K. Arumudasaamy N. (2016). Oncogene knockdown via active loading of small rnas into extracellular vesicles by sonication. *Cell. Mol. Bioeng.* 9 315–324. 10.1007/s12195-016-0457-4 27800035 PMC5084850

[B103] Lee C. Mitsialis S. A. Aslam M. Vitali S. H. Vergadi E. Konstantinou G. (2012). Exosomes mediate the cytoprotective action of mesenchymal stromal cells on hypoxia-induced pulmonary hypertension. *Circulation* 126, 2601–2611. 10.1161/circulationaha.112.114173 23114789 PMC3979353

[B104] Lee E.-Y. Choi D.-Y. Kim D.-K. Kim J.-W. Park J. O. Kim S. (2009). Gram-positive bacteria produce membrane vesicles: proteomics-based characterization of *Staphylococcus aureus*-derived membrane vesicles. *Proteomics* 9 5425–5436. 10.1002/pmic.200900338 19834908

[B105] Li J. W. Wie L. Han Z. Chen Z. (2019). Mesenchymal stromal cells-derived exosomes alleviate ischemia/reperfusion injury in mouse lung by transporting anti-apoptotic miR-21-5p. *Eur. J. Pharmacol.* 852 68–76. 10.1016/j.ejphar.2019.01.022 30682335

[B106] Li M. Yu D. Williams K. J. Liu M.-L. (2010). Tobacco smoke induces the generation of procoagulant microvesicles from human monocytes/macrophages. *Arterioscler. Thromb. Vasc. Biol.* 30 1818–1824. 10.1161/atvbaha.110.209577 20558816 PMC2939448

[B107] Lopes-Pacheco M. (2016). CFTR modulators: shedding light on precision medicine for cystic fibrosis. *Front. Pharmacol.* 7:275. 10.3389/fphar.2016.00275 27656143 PMC5011145

[B108] Lopes-Pacheco M. (2020). CFTR modulators: the changing face of cystic fibrosis in the era of precision medicine. *Front. Pharmacol.* 10:1662. 10.3389/fphar.2019.01662 PMC7046560 32153386

[B109] Lopes-Pacheco M. Bandeira E. Morales M. M. (2016). Cell-based therapy for silicosis. *Stem Cells Int.* 2016:5091838. 10.1155/2016/5091838 PMC4811211 27066079

[B110] Lopes-Pacheco M. Robba C. Rocco P. R. M. Pelosi P. (2020). Current understanding of the therapeutic benefits of mesenchymal stem cells in acute respiratory distress syndrome. *Cell Biol. Toxicol.* 36 83–102. 10.1007/s10565-019-09493-5 31485828 PMC7222160

[B111] Lopes-Pacheco M. Silva P. L. Cruz F. F. Battaglini D. Robba C. Pelosi P. (2021). Pathogenesis and multiple organ injury in COVID-19 and potential therapeutic strategies. *Front. Physiol.* 12:593223. 10.3389/fphys.2021.593223 PMC7876335 33584343

[B112] Lopes-Pacheco M. Xisto D. G. Ornellas F. M. Antunes M. A. Abreu S. C. Rocco P. R. M. (2013). Repeated administration of bone marrow-derived cells prevents disease progression in experimental silicosis. *Cell. Physiol. Biochem.* 32 1681–1694. 10.1159/000356603 24356399

[B113] Lötvall J. Hill A. F. Hochberg F. Buzás E. I. De Vizio D. Fardiner C. (2014). Minimal experimental requirements for definition of extracellular vesicles and their functions: a position statement from the International society for extracellular vesicles. *J. Extracell. Ves.* 3:26913. 10.3402/jev.v3.26913 25536934 PMC4275645

[B114] Makiguchi T. Yamada M. Yoshioka Y. Sugiura H. Kaorai A. (2016). Serum extracellular vesicular miR-21- 5p is a predictor of the prognosis in idiopathic pulmonary fibrosis. *Respir. Res.* 17:110. 10.1186/s12931-016-0427-3 PMC5011900 27596748

[B115] Mansouri N. Willis G. R. Fernandez-Gonzalez A. Reis M. Nassiri S. Mitsialis S. A. (2019). Mesenchymal stromal cell exosomes prevent and revert experimental pulmonar fibrosis through modulation of monocyte phenotypes. *JCI Insight* 4:e128060. 10.1172/jci.insight.128060 PMC6948760 31581150

[B116] Matthay M. A. Calfee C. S. Zhuo H. Thompson B. T. Wilson J. F. Levitt J. E. (2019). Treatment with allogeneic mesenchymal stromal cells for moderate to severe acute respiratory distress syndrome (START study), a randomised phase 2a safety trial. *Lancet Respir. Med.* 7 154–162. 10.1016/s2213-2600(18)30418-1 30455077 PMC7597675

[B117] Maumus M. Rozier P. Boulestreau J. Jorgensen C. Noël D. (2020). Mesenchymal stem cell-derived extracellular vesicles: opportunities and challenges for clinical translation. *Front. Bioeng. Biotechnol.* 8:997. 10.3389/fbioe.2020.00997 33015001 PMC7511661

[B118] McIntyre L. A. Stewart D. J. Mei S. H. J. Courtman D. Watpool I. Granton J. (2018). Cellular immunotherapy for septic shock. A phase I clinical trial. *Am. J. Respir. Crit. Care Med.* 197 337–347. 10.1164/rccm.201705-1006oc 28960096

[B119] Mendt M. Kamerkar S. Sugimoto H. McAndrews K. M. Wu C. C. Gagea M. (2018). Generation and testing of clinical-grade exosomes for pancreatic cancer. *JCI Insight* 3:e99263. 10.1172/jci.insight.99263 PMC5931131 29669940

[B120] Minciacchi V. R. Freeman M. R. Di Vizio D. (2015). Extracellular vesicles in cancer: exosomes, microvesicles and the emerging role of large oncosomes. *Semin. Cell Dev. Biol.* 40 41–51. 10.1016/j.semcdb.2015.02.010 25721812 PMC4747631

[B121] Mitri C. Xu Z. Bardin P. Corvol H. Touqui L. Tabary O. (2020). Novel anti-inflammatory approaches for cystic fibrosis lung disease. Identification of molecular targets and design of innovative therapies. *Front. Pharmacol.* 11:1096. 10.3389/fphar.2020.01096 32848733 PMC7396676

[B122] Mohammadipoor A. Antebi B. Batchinsky A. I. Cancion L. C. (2018). Therapeutic potential of products derived from mesenchymal stem/stromal cells in pulmonary disease. *Respir. Res.* 19:218. 10.1186/s12931-018-0921-x PMC6234778 30413158

[B123] Mokarizadeh A. Delirezh N. Morshedi A. Mosayebi G. Darshid A.-A. Mardani K. (2012). Microvesicles derived from mesenchymal stem cells: potent organelles for induction of tolerogenic signaling. *Immunol. Lett.* 147 47–54. 10.1016/j.imlet.2012.06.001 22705267

[B124] Monsel A. Zhu Y. G. Gennai S. Hao Q. Hu S. Rouby J.-J. (2015). Therapeutic effects of human mesenchymal stem cell-derived microvesicles in severe pneumonia in mice. *Am. J. Respir. Crit. Care Med.* 192 324–336. 10.1164/rccm.201410-1765oc 26067592 PMC4584251

[B125] Monsel A. Zhu Y. G. Gennai S. Hao Q. Liu J. Lee J. W. (2014). Cell-based therapy for acute organ injury: preclinical evidence and ongoing clinical trials using mesenchymal stem cells. *Anesthesiology* 121 1099–1121. 25211170 10.1097/ALN.0000000000000446 PMC4206665

[B126] Morrison T. J. Jackson M. V. Cunningham E. K. Kissenpfenning A. McAuley D. F. O’Kane C. M. (2017). Mesenchymal stromal cells modulate macrophages in clinically relevant lung injury models by extracellular vesicle mitochondrial transfer. *Am. J. Respir. Crit. Care Med.* 196 1275–1286. 10.1164/rccm.201701-0170oc 28598224 PMC5694830

[B127] O’Farrell H. E. Yang I. A. (2019). Extracellular vesicles in chronic obstructive pulmonary disease (COPD). *J. Thorac. Dis.* 11 (Suppl. 17), S2141–S2154. 31737342 10.21037/jtd.2019.10.16 PMC6831918

[B128] Orme J. Jr. Romney J. S. Hopkins R. O. Pope D. Chan K. J. Thomsen G. (2003). Pulmonary function and health-related quality of life in survivors of acute respiratory distress syndrome. *Am. J. Respir. Crit. Care Med.* 167 690–694. 10.1164/rccm.200206-542oc 12493646

[B129] Padilha G. A. Henriques I. Lopes-Pacheco M. Abreu S. C. Oliveira M. V. Morales M. M. (2015). Therapeutic effects of LASSBio-596 innan elastase-induced mouse model of emphysema. *Front. Physiol.* 6:267. 10.3389/fphys.2015.00267 26483698 PMC4588117

[B130] Pan B. T. Teng K. Wu C. Adam M. Johnstone R. M. (1985). Electron microscopic evidence for externalization of the transferrin receptor in vesicular form in sheep reticulocytes. *J. Cell Biol.* 101 942–948. 10.1083/jcb.101.3.942 2993317 PMC2113705

[B131] Papierniak E. S. Lowenthal D. T. Harman E. (2013). Novel therapies in asthma: leukotriene antagonists, biologic agents, and beyond. *Am. J. Ther.* 20 79–103. 10.1097/mjt.0b013e31826915c2 23299231

[B132] Park J. Kim S. Lim H. Liu A. Hu S. Lee J. (2019). Therapeutic effects of human mesenchymal stem cell microvesicles in an ex vivo perfused human lung injured with severe E. coli pneumonia. *Thorax* 74 43–50. 10.1136/thoraxjnl-2018-211576 30076187 PMC6295323

[B133] Phinney D. G. Di Giuseppe M. Njah J. Sala E. Shiva S. St Croix C. M. (2015). Mesenchymal stem cells use extracellular vesicles to outsource mitophagy and shuttle microRNAs. *Nat. Commun.* 6:8472. 10.1038/ncomms9472 PMC4598952 26442449

[B134] Poggio H. A. Antunes M. A. Rocha N. N. Kitoko J. Z. Morales M. M. Olsen P. C. (2018). Impact of one versus two doses of mesenchymal stromal cells on lung and cardiovascular repair in experimental emphysema. *Stem Cell Res. Ther.* 9:293. 10.1186/s13287-018-1043-6 PMC6225700 30409216

[B135] Popowski K. Lutz H. Hu S. George A. Phuong-Uyen D. Cheng K. (2020). Exosome therapeutics for lung regenerative medicine. *J. Extracell. Ves.* 9:1785161. 10.1080/20013078.2020.1785161 32944172 PMC7480570

[B136] Porzionato A. Zaramella P. Dedja A. Guidolin D. Van Wemmel K. Macchi V. (2019). Intratracheal administration of clinical-grade mesenchymal stem cell-derived extracellular vesicles reduces lung injury in a rat model of bronchopulmonary dysplasia. *Am. J. Physiol. Lung Cell Mol. Physiol.* 316 L6–L19. 30284924 10.1152/ajplung.00109.2018

[B137] Prado N. Marazuela E. G. Segura E. Fernández-García H. Villalba M. Théry C. (2008). Exosomes from bronchoalveolar fluid of tolerized mice prevent allergic reaction. *J. Immunol.* 181 1519–1525. 10.4049/jimmunol.181.2.1519 18606707

[B138] Pua H. H. Happ H. C. Gray C. J. Mar D. J. Chiou N.-T. Hesse L. E. (2019). Increased hematopoietic extracellular RNAs and vesicles in the lung during allergic airway responses. *Cell Rep.* 26 933–944. 10.1016/j.celrep.2019.01.002 30673615 PMC6365014

[B139] Qazi K. R. Torregrosa Paredes P. Dahlberg B. Grunewald J. Eklund A. Gabrielsson S. (2010). Proinflammatory exosomes in bronchoalveolar lavage fluid of patients with sarcoidosis. *Thorax* 65 1016–1024. 10.1136/thx.2009.132027 20880880

[B140] Qian X. Xu C. Fang S. Zhao P. Wang Y. Liu H. (2016). Exosomal MicroRNAs derived from umbilical mesenchymal stem cells inhibit Hepatitis C virus infection. *Stem Cells Transl. Med.* 5 1190–1203. 10.5966/sctm.2015-0348 27496568 PMC4996444

[B141] Raposo G. Nijman H. W. Stoorvogel W. Liejendekker R. Harding C. V. Melief C. J. (1996). B lymphocytes secrete antigen-presenting vesicles. *J. Exp. Med.* 183 1161–1172. 10.1084/jem.183.3.1161 8642258 PMC2192324

[B142] Ray A. Oriss T. B. Wenzel S. E. (2015). Emerging molecular phenotypes of asthma. *Am. J. Physiol. Cell. Mol. Physiol.* 308 L130–L140. 10.1152/ajplung.00070.2014 PMC4338947 25326577

[B143] Rodrigues M. Fan J. Lyon C. Wan M. Hu Y. (2018). Role of extracellular vesicles in viral and bacterial infections: pathogenesis, diagnostics, and therapeutics. *Theranostics* 8 2709–2727. 10.7150/thno.20576 29774070 PMC5957004

[B144] Roy K. Hamilton D. J. Munson G. P. Fleckenstein J. M. (2011). Outer membrane vesicles induce immune responses to virulence proteins and protect against colonization by enterotoxigenic *Escherichia coli*. *Clin. Vaccine Immunol.* 18 1803–1808. 10.1128/cvi.05217-11 21900530 PMC3209013

[B145] Sarkar A. Mitra S. Mehta S. Raices R. Wewers M. D. (2009). Monocyte derived microvesicles deliver a cell death message via encapsulated caspase-1. *PLoS One* 4:e7140. 10.1371/journal.pone.0007140 19779610 PMC2744928

[B146] Sauleda J. Núnez B. Saula E. Soriano J. B. (2018). Idiopathic pulmonary fibrosis: epidemiology, natural history, phenotypes. *Med. Sci. (Basel).* 6:110. 10.3390/medsci6040110 30501130 PMC6313500

[B147] Savarimuthu Francis S. M. Davidson M. R. Tan M. E. Wright C. M. Clarke B. E. Duhig E. E. (2014). MicroRNA-34c is associated with emphysema severity and modulates SERPINE1 expression. *BMC Genom.* 15:88. 10.1186/1471-2164-15-88 24479666 PMC3922660

[B148] Sengupta V. Sengupta S. Lazo A. Woods P. Nolan A. Bremer N. (2020). Exosomes derived from bone marrow mesenchymal stem cells as treatment for severe COVID-19. *Stem Cells Dev.* 29 747–754. 10.1089/scd.2020.0080 32380908 PMC7310206

[B149] Serban K. A. Rezania S. Petrusca D. N. Poirier C. Cao D. Justice M. J. (2016). Structural and functional characterization of endothelial microparticles released by cigarette smoke. *Sci. Rep.* 6:31596. 10.1038/srep31596 PMC4987682 27530098

[B150] Sethi S. Murphy T. F. (2008). Infection in the pathogenesis and course of chronic obstructive pulmonary disease. *N. Engl. J. Med.* 359 2355–2365. 19038881 10.1056/NEJMra0800353

[B151] Shentu T.-P. Huang T.-S. Cernelc-Kohan M. Chan J. Wong S. S. Espinoza C. R. (2017). Thy-1 dependent uptake of mesenchymal stem cell-derived extracellular vesicles blocks myofibroblastic differentiation. *Sci. Rep.* 7:18052. 10.1038/s41598-017-18288-9 PMC5741716 29273797

[B152] Silva J. D. de Castro L. L. Braga C. L. Oliveira G. P. Trivelin A. S. Barbosa-Junior C. M. (2019). Mesenchymal stromal cells are more effective than their extracellular vesicles at reducing lung injury regardless of acute respiratory distress syndrome etiology. *Stem Cells Int.* 2019 8262849. 10.1155/2019/8262849 PMC6720722 31531026

[B153] Silva J. D. Lopes-Pacheco M. Paz A. H. R. Cruz F. F. Melo E. B. de Oliveira M. V. (2018). Mesenchymal stem cells from bone marrow, adipose tissue, and lung tissue differentially mitigate lung and distal organ damage in experimental acute respiratory distress syndrome. *Crit. Care Med.* 46 e132–e140. 29116998 10.1097/CCM.0000000000002833

[B154] Simoncini S. Njock M.-S. Robert S. Camoin-Jau L. Sampol J. Harlé J.-R. (2009). TRAIL/Apo2L mediates the release of procoagulant endothelial microparticles induced by thrombin in vitro: a potential mechanism linking inflammation and coagulation. *Circ. Res.* 104 943–951. 10.1161/circresaha.108.183285 19265041

[B155] Singh D. Agusti A. Anzueto A. Barners P. J. Bourbeau J. Celli B. R. (2019). Global strategy for the diagnosis, management, and prevention of chronic obstructive lung disease: the GOLD science committee report 2019. *Eur. Respir. J.* 53 1900164. 10.1183/13993003.00164-2019 30846476

[B156] Song Y. Dou H. Li X. Zhao X. Li Y. Liu D. (2017). Exosomal miR-146a contributes to the enhanced therapeutic efficacy of interleukin-1β-primed mesenchymal stem cells against sepsis. *Stem Cells* 35 1208–1221. 10.1002/stem.2564 28090688

[B157] Stokes J. R. Casale T. B. (2016). Characterization of asthma endotypes: implications for therapy. *Ann. Allergy Asthma Immunol.* 117 121–125. 10.1016/j.anai.2016.05.016 27499539

[B158] Sun D. Zhuang X. Xiang X. Liu Y. Zhang S. Liu C. (2010). A novel nanoparticle drug delivery system: the anti-inflammatory activity of curcumin is enhanced when encapsulated in exosomes. *Mol. Ther.* 18 1606–1614. 10.1038/mt.2010.105 20571541 PMC2956928

[B159] Tabary O. Corvol H. Boncoeur E. Chadelat K. Fitting C. Cavaillon J. M. (2006). Adherence of airway neutrophils and inflammatory response are increased in CF airway epithelial cell-neutrophil interactions. *Am. J. Physiol. Lung Cell Mol. Physiol.* 290 L588–L596. 16272177 10.1152/ajplung.00013.2005

[B160] Takahashi T. Kobayashi S. Fujino N. Suzuki T. Ota C. He M. (2012). Increased circulating endothelial microparticles in COPD patients: a potential bioindicator for COPD exacerbation susceptibility. *Thorax* 67:1067. 10.1136/thoraxjnl-2011-201395 22843558

[B161] Tan D. B. A. Armitage J. Teo T. H. Ong N. E. Shin H. Moodley Y. P. (2017). Elevated levels of circulating exosome in COPD patients are associated with systemic inflammation. *Respir. Med.* 132 261–264. 10.1016/j.rmed.2017.04.014 28476471

[B162] Tan J. L. Lau S. N. Leaw B. Nguyen H. P. T. Salamonsen L. A. Saad M. I. (2018). Annion epithelial cell-derived exosomes restric lung injury and enhance endogenous lung repair. *Stem Cells Transl. Med.* 7 180–196. 10.1002/sctm.17-0185 29297621 PMC5788876

[B163] Tang X. D. Shi L. Monsel A. Li X.-Y. Zhu H.-L. Zhu Y.-G. (2017). Mesenchymal stem cell microvesicles attenuate acute lung injury in mice partly mediated by Ang-1 mRNA. *Stem Cells* 35 1849–1859. 10.1002/stem.2619 28376568

[B164] Tauro B. J. Greening D. W. Mathias R. A. Ji H. Mathivanan S. Scott A. M. (2012). Comparison of ultracentrifugation, density gradient separation, and immunoaffinity capture methods for isolating human colon cancer cell line LIM1863-derived exosomes. *Methods* 56 293–304. 10.1016/j.ymeth.2012.01.002 22285593

[B165] Taylor D. D. Shah S. (2015). Methods of isolating extracellular vesicles impact down-stream analyses of their cargoes. *Methods* 87 3–10. 10.1016/j.ymeth.2015.02.019 25766927

[B166] Tetta C. Bruno S. Fonsato V. Deregibus M. C. Camussi G. (2011). The role of microvesicles in tissue repair. *Organogenesis* 7 105–115. 10.4161/org.7.2.15782 21572253 PMC3142447

[B167] Thery C. Amigorena S. Raposo G. Clayton A. (2006). Isolation and characterization of exosomes from cell culture supernatants and biological fluids. *Curr. Protoc. Cell Biol.* 3:Unit3.22. 10.1002/0471143030.cb0322s30 18228490

[B168] Theìry C. Witwer K. W. Aikawa E. Alcaraz M. J. Anderson J. D. Andriantsitohaina R. (2018). Minimal information for studies of extracellular vesicles 2018 (MISEV2018), a position statement of the international society for extracellular vesicles and update of the MISEV2014 guidelines. *J. Extracell. Ves.* 7:1535750. 10.1080/20013078.2018.1535750 PMC6322352 30637094

[B169] Ti D. Hao H. Tong C. Liu J. Dong L. Zheng J. (2015). LPS-preconditioned mesenchymal stromal cells modify macrophage polarization for resolution of chronic inflammation via exosome-suttled let-7b. *J. Transl. Med.* 13:308. 10.1186/s12967-015-0642-6 PMC4575470 26386558

[B170] Tomasoni S. Longaretti L. Rota C. Mirigi M. Conti S. Gotti E. (2013). Transfer of growth factor receptor mRNA via exosomes unravels the regenerative effect of mesenchymal stem cells. *Stem Cells Dev.* 22 772–780. 10.1089/scd.2012.0266 23082760 PMC3578372

[B171] Torregrosa Paredes P. Esser J. Admyre C. Nord M. Rahman Q. K. Lukic A. (2012). Bronchoalveolar lavage fluid exosomes contribute to cytokine and leukotriene production in allergic asthma. *Allergy* 67 911–919. 10.1111/j.1398-9995.2012.02835.x 22620679

[B172] Trajkovic K. Hsu C. Chiantia S. Rajendran L. Wenzel D. Wieland F. (2008). Ceramide triggers budding of exosome vesicles into multivesicular Endosomes. *Science* 319 1244–1247. 10.1126/science.1153124 18309083

[B173] Varkouhi A. K. Jerkic M. Ormesher L. Gagnon S. Goyal S. Rabani R. (2019). Extracellular vesicles from interferon-gamma-primed human umbilical cord mesenchymal stromal cells reduce *Escherichia coli*-induced acute lung injury in rats. *Anesthesiology* 130 778–790. 10.1097/aln.0000000000002655 30870158

[B174] Vituret C. Gallay K. Confort M.-P. Ftaich N. Matei C. I. Archer F. (2016). Transfer of the cystic fibrosis transmembrane conductance regulator to human cystic fibrosis cells mediated by extracellular vesicles. *Hum. Gene Ther.* 27 166–183. 10.1089/hum.2015.144 26886833

[B175] Wang H. Zheng R. Chen Q. Shao J. Yu J. Hu S. (2017). Mesenchymal stem cells microvesicles stabilizes endotelial barrier function partly mediated by hepatocyte growth factor (HGF). *Stem Cell Res. Ther.* 8:211. 10.1186/s13287-017-0662-7 PMC5623961 28969681

[B176] Wang J. Huang R. Xu Q. Zheng G. Qiu G. Ge M. (2020). Mesenchymal stem cell-derived extracellular vesicles alleviate acute lung injury via transfer of miR-27a-3p. *Crit. Care Med.* 48 e599–e610. 32317602 10.1097/CCM.0000000000004315

[B177] Waszak P. Alphonse R. Vadivel A. Ionescu L. Eaton F. Thébaud B. (2012). Preconditioning enhances the paracrine effect of mesenchymal stem cells in preventing oxygen-induced neonatal lung injury in rats. *Stem Cells Dev.* 21 2789–2797. 10.1089/scd.2010.0566 22533467

[B178] Wiklander O. P. B. Brennan M. Á Lötvall J. Breakefield X. O. El Andaloussi S. (2019). Advances in therapeutic applications of extracellular vesicles. *Sci. Transl. Med.* 11:eaav8521. 10.1126/scitranslmed.aav8521 31092696 PMC7104415

[B179] Willis G. R. Fernandez-Gonzalez A. Anastas J. Vitali S. H. Liu X. Ericsson M. (2018). Mesenchymal Stromal cell Exosomes ameliorate experimental bronchopulmonary Dysplasia and restore lung function through macrophage immunomodulation. *Am. J. Respir. Crit. Care Med.* 197 104–116. 10.1164/rccm.201705-0925oc 28853608 PMC5765387

[B180] Witwer K. W. Soekmadji C. Hill A. F. Wauben M. H. Buzás E. I. De Vizio D. (2017). Updating the MISEV minimal requirements for extracellular vesicle studies: building bridges to reproducibility. *J. Extracell. Ves.* 6:1396823. 10.1080/20013078.2017.1396823 29184626 PMC5698937

[B181] Witwer K. W. Van Balkom B. W. M. Bruno S. Choo A. Dominici M. Gimona M. (2019). Defining mesenchymal stromal cell (MSC)-derived small extracellular vesicles for therapeutic applications. *J. Extracell. Ves.* 29:1609206. 10.1080/20013078.2019.1609206 31069028 PMC6493293

[B182] Wu X. Liu Z. Hu L. Gu W. Zhu L. (2018). Exosomes derived from endothelial progenitor cells ameliorate acute lung injury by transferring miR-126. *Exp. Cell Res.* 370 13–23. 10.1016/j.yexcr.2018.06.003 29883714

[B183] Xu H. Ling M. Xue J. Dai X. Sun Q. Chen C. (2018). Exosomal microRNA-21 derived from bronchial epithelial cells is involved in aberrant epithelium-fibroblast cross-talk in COPD induced by cigarette smoking. *Theranostics* 8 5419–5433. 10.7150/thno.27876 30555555 PMC6276085

[B184] Yi X. Wei X. Lv H. Na Y. Li L. Lu P. (2019). Exosomes derived from microRNA-30b-3p-overexpressing mesenchymal stem cells protect against lipopolysaccharide-induced acute lung injury by inhibiting SAA3. *Exp. Cell Res.* 383:111454. 10.1016/j.yexcr.2019.05.035 31170401

[B185] Yu Q. Wang D. Wen X. Tang X. Qi D. He J. (2020). Adipose-derived exosomes protect the pulmonary endothelial barrier in ventilator-induced lung injury by inhibiting the TRPV4/Ca^2+^ signaling pathway. *Am. J. Physiol. Lung Cell Mol Physiol.* 318 L723–L741. 32073873 10.1152/ajplung.00255.2019 PMC7191475

[B186] Zhang B. Tian X. Hao J. Xu G. Zhang W. (2020). Mesenchymal stem cell-derived extracellular vesicles in tissue regeneration. *Cell Transpl.* 29:0963689720908500. 10.1177/0963689720908500 32207341 PMC7444208

[B187] Zhang B. Yin Y. Lai R. C. Tan S. S. Choo A. B. H. Lim S. K. (2014). Mesenchymal stem cells secrete immunologically active exosomes. *Stem Cells Dev.* 23 1233–1244. 10.1089/scd.2013.0479 24367916

[B188] Zhang C. Wang P. Mohammed A. Zhou Z. Zhang S. Ni S. (2020). Function of Adiopse-derived mesenchymal stem cells in monocrotaline-induced pulmonary arterial hypertension through miR-191 via regulation of BMPR2. *Biomed. Res. Int.* 2019:2858750. 10.1155/2019/2858750 PMC6500697 31119161

[B189] Zhang D. Lee H. Zhu Z. Minhas J. K. Jin Y. (2017). Enrichment of selective miRNAs in exosomes and delivery of exosomal miRNAs in vitro and in vivo. *Am. J. Physiol. Lung Cell Mol. Physiol.* 312 L110–L121. 27881406 10.1152/ajplung.00423.2016 PMC5283929

[B190] Zhao S. Wehner R. Bornhauser M. Wassmuth R. Bachmann M. Schmitz M. (2010). Immunomodulatory properties of mesenchymal stromal cells and their therapeutic consequences for immune-mediated disorders. *Stem Cells Dev.* 19 607–614. 10.1089/scd.2009.0345 19824807

[B191] Zheng G. Huang L. Tong H. Shu Q. Hu Y. Ge M. (2014). Treatment of acute respiratory distress syndrome with allogeneic adipose-derived mesenchymal stem cells: a randomized, placebo-controlled pilot study. *Respir. Res.* 15:39. 10.1186/1465-9921-15-39 24708472 PMC3994204

[B192] Zhou Y. Li P. Goodwin A. J. Cook J. A. Halushka P. V. Chang E. (2019). Exosomes from endothelial progenitor cells improve outcomes of the lipopolysaccharide-induced acute lung injury. *Crit. Care* 23:44. 10.1186/s13054-019-2339-3 PMC6373158 30760290

[B193] Zhu Y. G. Feng X. M. Abbott J. Fang X.-H. Hao Q. Monsel A. (2014). Human mesenchymal stem cell microvesicles for treatment of *Escherichia coli* endotoxin-induced acute lung injury in mice. *Stem Cells* 32 116–125. 10.1002/stem.1504 23939814 PMC3947321

[B194] Zulueta A. Colombo M. Peli V. Falleni M. Tosi D. Ticciardi M. (2018). Lung mesenchymal stem cells-derived extracellular vesicles attenuate the inflammatory profile of cystic fibrosis epithelial cells. *Cell Signal.* 51 110–118. 10.1016/j.cellsig.2018.07.015 30076968

